# Clusterin Inhibits Neuronal Ferroptosis via the PI3K-AKT-mTOR-SREBP1 Axis to Promote Functional Recovery after Spinal Cord Injury

**DOI:** 10.7150/ijbs.124291

**Published:** 2026-01-15

**Authors:** Senyu Yao, Ziming Wang, Xiaokang Wang, Yangfan Yu, Xu Huang, Liqi Chen, Zhenming Tian, Bin Liu, Yang Yang, Mao Pang, Limin Rong

**Affiliations:** 1 Department of Spine Surgery, The Third Affiliated Hospital of Sun Yat-Sen University, Guangzhou, 510630, China.; 2 National Medical Products Administration (NMPA) Key Laboratory for Quality Research and Evaluation of Cell Products, The Third Affiliated Hospital of Sun Yat-Sen University, Guangzhou, 510630, China.; 3 Guangdong Engineering Technology Research Center of Minimally Invasive Spine Surgery, The Third Affiliated Hospital of Sun Yat-Sen University, Guangzhou, 510630, China.

**Keywords:** clusterin (CLU), neuronal ferroptosis, spinal cord injury (SCI), PI3K-AKT-mTOR pathway, SREBP1-SCD1 axis, ACSL4

## Abstract

Neuronal ferroptosis is considered as a key mechanism contributing to neurological deficits during the secondary injury phase following spinal cord injury (SCI). Clusterin (CLU), a stress-responsive protein, has been reported to exert neuroprotective effects and promote neuronal survival in central nervous system injuries. However, its specific role in neuronal ferroptosis remains unclear. Here, we demonstrate that both exogenous recombinant CLU protein and endogenous CLU overexpression significantly inhibit neuronal ferroptosis, as evidenced by reduced lipid peroxidation, decreased iron accumulation, preserved mitochondrial integrity, and modulation of ferroptosis-related genes (upregulation of GPX4/xCT and downregulation of ACSL4). Mechanistically, CLU activates the PI3K-AKT-mTOR pathway, subsequently regulating the SREBP1-SCD1 lipid metabolism axis to suppress ACSL4-mediated lipid peroxidation. Furthermore, AAV-mediated CLU overexpression effectively mitigates pathological damage and significantly enhances motor function recovery in SCI mice. In conclusion, this study reveals a novel mechanism whereby CLU promotes SCI repair by inhibiting neuronal ferroptosis via the PI3K-AKT-mTOR-SREBP1 axis, indicating its therapeutic potential for ferroptosis-targeted neuroprotective strategies.

## Introduction

Spinal cord injury (SCI) is a devastating neurological trauma causing profound physical, psychological, and socioeconomic burdens^1^. Current therapies offer primarily symptomatic relief, but critically fail to achieve significant functional neurological recovery^2^-^4^. This stark therapeutic limitation underscores the urgent need to elucidate the underlying pathophysiology of SCI, particularly the progressive secondary injury cascade, characterized by neuronal and glial loss, which is driven by processes such as oxidative stress, neuroinflammation, and excitotoxicity^5^,^6^.

The compromised blood-spinal cord barrier (BSCB) following spinal cord injury (SCI) initiates a detrimental cascade within the spinal cord microenvironment, characterized by ischemia and excessive inflammatory infiltration^7^. Crucially, this ischemia triggers massive free radical generation, which attacks polyunsaturated fatty acids (PUFAs) in cell membranes to initiate an iron-dependent lipid peroxidation chain reaction^8^. The process generates highly toxic end products, such as malondialdehyde (MDA) and 4-hydroxynonenal (4-HNE), that damage membranes and organelles, ultimately exacerbating neurological deficits^9^.

Programmed cell death (PCD) mechanisms are pivotal executors of this secondary damage^10^, with the disrupted post-SCI microenvironment activating diverse forms including apoptosis, necroptosis, pyroptosis, autophagy, and ferroptosis^11^-^15^. Among these, ferroptosis has emerged as a principal contributor due to its intrinsic link to iron-dependent lipid peroxidation^16^. Biochemically, ferroptosis is defined by the insufficiency of the lipid repair enzyme GPX4, coupled with the essential activity of acyl-CoA synthetase long-chain family member 4 (ACSL4). ACSL4 drives ferroptotic sensitivity by enriching cellular membranes with peroxidizable PUFAs, thereby establishing the lipid substrate for this deleterious process^17^.

Compelling evidence firmly establishes ferroptosis within SCI pathogenesis. BSCB rupture leads to hemorrhage, with subsequent hemoglobin degradation releasing free iron and causing significant iron accumulation at the lesion site^18^. This is accompanied by elevated levels of lipid peroxidation markers (e.g., MDA, 4-HNE), which correlate with functional impairment. Critically, ferroptosis-specific interventions, such as the iron chelator deferoxamine (DFO), selenium (to enhance GPX4 activity), or mitochondrial transfer via MSC therapy, effectively mitigate lipid peroxidation and promote functional recovery^19^-^21^. Furthermore, ultrastructural analyses consistently reveal hallmark ferroptotic morphology, notably shrunken mitochondria with condensed membranes and lost cristae, in both neurons and glia^22^. Therefore, targeted inhibition of neuronal ferroptosis represents a promising neuroprotective strategy, although its precise regulatory mechanisms require further elucidation.

Intriguingly, Clusterin (CLU), a stress-responsive chaperone, exhibits functional properties that position it as a potential key modulator of ferroptosis^23^. While constitutively expressed at low levels in the healthy CNS, CLU is markedly upregulated following various CNS injuries, including traumatic brain injury (TBI) and ischemia^24^. Traditionally associated with neuroprotection through functions like clearing misfolded proteins and modulating apoptosis^25^,^26^, crucially, emerging evidence highlights CLU's direct capacity to combat oxidative stress and suppress lipid peroxidation^27^-^30^. In neuronal cell lines, elevated reactive oxygen species and lipid peroxidation directly induce CLU expression, suggesting an endogenous defense response^27^,^28^. Significantly, *in vivo* studies demonstrate that CLU overexpression or exogenous administration markedly attenuates levels of key oxidative stress markers, including 4-HNE, a terminal executor of lipid peroxidation central to ferroptosis, and reduces cell death in models of cerebral ischemia and TBI^29^,^30^. Despite these compelling connections, fundamental questions remain unanswered: the specific function of CLU in SCI, its direct relationship with ferroptosis, and the precise molecular mechanisms underlying its potential protective effects. Moreover, the spatiotemporal dynamics of CLU expression and its potential interplay with key ferroptotic drivers like ACSL4 during SCI progression have not been systematically investigated.

In this study, we bridge these knowledge gaps by delineating the temporal expression and functional interplay between CLU and ACSL4 after SCI. We demonstrate that CLU confers neuroprotection primarily by suppressing ACSL4-triggered neuronal ferroptosis. Mechanistically, CLU activates the PI3K-AKT-mTOR signaling axis to promote SREBP1-SCD1-mediated monounsaturated fatty acid (MUFA) synthesis, thereby countering lethal phospholipid peroxidation. Our findings thus establish a mechanistic foundation for novel CLU-based therapeutic strategies against SCI.

## Materials and Methods

### Animals

Animal use and all experiments involving animals were approved by the Ethical Committee of Sun Yat-sen University, China (SYSU-IACUC-2023-000652). Only female mice were used throughout the experiment. C57BL/6J mice aged 8 weeks and weighing 15-20 g were purchased from Guangdong GemPharmatech. All the animals were provided free access to food and water and kept in a colony room under conditions of constant temperature (25°C), humidity (70%), and lighting (12 h light/12 h dark cycle) in the Sun Yat-sen University Animal Center.

### Spinal cord injury surgeries

The procedure of T10 crush injury of spinal cord was performed according to previous established protocol^19^. In brief, C57BL/6J mice were anesthetized by tribromethanol (T48402, Sigma-Aldrich) and a 2 cm midline incision was made over the dorsal thoracic vertebrae, followed by a T9-T10 laminectomy to fully expose the spinal cord. The Dumont micro forceps (11295-00, F.S.T) was used to clamp the T10 spinal cord for complete transverse injury. Tips of forceps were inserted along bilateral sides of the spinal cord and touched the vertebrae on the ventral side to cover the whole spinal cord. The spinal cord was then crushed for 5 s with forceps. This procedure left an obvious trace of hemorrhagic injury on the surface of the T10 spinal cord. The muscle layers were sutured and wound clips were used to close the skin. Mice were placed on a warming blanket after surgery until thoroughly awake. Dysfunctional bladders were manually emptied once a day until their autonomous urination function recovered.

### AAV injection

The mice were anesthetized with isoflurane (R510-22, RWD) and spine-fixed using a stereotaxic frame (RWD). AAV titers were adjusted to 10^13^ copies/mL for injection and each mouse was injected with 250 nL. Following T9-T10 laminectomy, a micropipette attached to microliter syringe (80135, Hamilton) was used to deliver pAAV9-CMV-EGFP-3xFLAG-WPRE or pAAV9-CMV-Clu-P2A-EGFP-3xFLAG-WPRE (constructed by OBiO Technology Corp.,Ltd., Shanghai, China) into T10 spinal cord segment. Injection coordinates were set 0.3 mm lateral to the midline with two depths (-0.4 mm and -0.8 mm ventral to the spinal cord surface).

### Cell culture

HT22 cells (mouse primary hippocampal neurons) were purchased from Procell (CL-0697, China) and grown in the high-glucose Dulbecco's Modified Eagle Medium (DMEM; Gibco) supplemented with 10% fetal bovine serum (FBS; Invitrogen), 100 IU/mL penicillin-streptomycin (eLGbio). All the cells were cultured in a humidified 5% CO_2_ - 95% air environment at 37 °C.

For *in vitro* drug treatment experiments, RAS-selective-lethal-3 (RSL3, HY-100218A, MCE) was used to induce intracellular ferroptosis in HT22 cells with 2.5, 5 or 10 μM.

For fluorescence staining of cell, 10 μM BODIPY 581/591 C11 (C1022, Beyotime) and 1 μM FerroOrange (HY-D1913, MCE) were used according to the manufacturers' protocols respectively. Briefly, HT22 cells were incubated with indicated fluorescent dyes in culture media and the excess dye was washed away with PBS three times. Subsequently, inverted fluorescence microscopy or confocal microscopy was used to obtain fluorescence images.

### Lentivirus vector transfection

For loss of CLU function, LV (pSLenti-U6-shRNA (Clu)-CMV-EGFP-F2A-Puro-WPRE) with CLU shRNA or LV (pSLenti-U6-shRNA(NC2)-CMV-EGFP-F2A-Puro-WPRE) with NC shRNA were used to transfect HT22 according to the manufacturer's protocol. 1×10^5^ cells were transfected by virus and filtered by 2 μg/mL puromycin after 72 h.

For CLU overexpression, LV (pcSLenti-EF1-EGFP-P2A-Puro-CMV-Clu-3xFLAG-WPRE) with full-length CLU or LV (pcSLenti-EF1-EGFP-P2A-Puro-CMV-MCS-3xFLAG-WPRE) with empty vector were transfected into HT22 as mentioned above. Lentivirus vectors were constructed by OBiO Technology Corp., Ltd. The target sequences of shRNAs used were described in **Supplementary Table 4**.

### Cell viability

The viability of HT22 cells was detected by a CCK-8 kit (GK10001, GLPBIO) in a 96-well plate. Cells were seeded at a concentration of 1 × 10^3^ per well. After RSL3 treatment, 10 µL of CCK-8 reagent was added to each well at 37 °C for 2 h, and the absorbance of each well was measured at 450 nm using a microplate reader.

### RNA isolation and real-time quantitative PCR

Total RNA was extracted from tissue or cells using the Trizol (T9424, Sigma-aldrich) and complementary DNA (cDNA) was synthesized using a StarScript II First-strand cDNA Synthesis Kit-II (A214, GenStar) according to the manufacturers' protocols. In brief, concentration of total RNA was quantified with a NanoDrop 8000 spectrophotometer and 1 µg of total RNA was used for reverse transcription. Target mRNA levels were quantified by performing qRT-PCR with a 2× RealStar Fast SYBR qPCR Mix (A301, GenStar), as described by the manufacturers' protocols. The thermocycling protocol for all experiments was 40 cycles of denaturation at 95 °C for 15 s, annealing at 60 °C for 15 s, and extension at 72 °C for 30 s. All samples were run in triplicate and target mRNA levels were normalized to those of Actin. The primers designed and used for qPCR are described in **Supplementary Table 1**.

### Western blot

Tissue was cut into fragments and homogenized by adding cold RIPA lysis buffer (P0013, Beyotime) with 100× protease inhibitor and phosphatase inhibitor cocktails. The homogenate was incubated on ice for 30 min to allow complete lysis, and supernatant was centrifuged at 10,000 g for 10 min to pellet cellular debris, and the resulting supernatant was collected for subsequent analysis.

Cell suspensions were collected and washed three times with cold phosphate-buffered saline. Then, the cells were lysed in 1× RIPA buffer with 100× protease inhibitor and phosphatase inhibitor cocktails for at least 30 min, and centrifuged at 10,000 g for 10 min at 4 °C to remove cell debris. The protein supernatant was aspirated and mixed with 4× loading buffer (Bio-RED). After total protein concentration was measured using BCA Protein Assay Kit (Thermo Scientific), equal amounts of total proteins were resolved by 10% SDS-PAGE (Bio-Rad) and then electrotransferred to a 0.2 μm pore-sized polyvinylidene difluoride membrane (Millipore, Darmstadt). After blocking with 5% BSA, the membrane was incubated with the primary antibodies at 4°C overnight, followed by incubation with HRP-conjugated secondary antibodies for 1 h at room temperature. The primary and secondary antibodies used can be found in the **Supplementary Table 2**. The chemiluminescent substrate (Millipore) was used to detect the signal intensity. Bands from at least three independent blots were quantified using the Image J software.

### Immunocytochemistry and Immunofluorescence

Cells were seeded onto coverslips and treated accordingly for each experiment. If necessary, fixation of cells occurred in 4% paraformaldehyde (PFA) for 15 min. Subsequently, cells were incubated with the primary antibodies overnight at 4 °C, followed by incubation with secondary antibodies away from light for 1 h at room temperature. The primary and secondary antibodies used were described in **Supplementary Table 2**. Images were acquired using LSM 980 confocal microscope (Zeiss).

Spinal cord samples were fixed in 4% PFA and then embedded in OCT compound for subsequent sectioning. Each tissue section was incubated with the primary antibodies overnight at 4 °C, followed by incubation with secondary antibodies away from light for 1 h at room temperature. The primary and secondary antibodies used were described in **Supplementary Table 2**. Images were acquired using LSM 980 confocal microscope (Zeiss).

### Histological analysis

H&E, Nissl and Prussian blue co-DAB staining were carried out for histological analysis. After sacrifice, mouse T10 spinal cord segment that involves the injury epicenter was dissected, perfused with 4% PFA and prepared for the paraffin embedding or saturation in 30% sucrose for 24 h for frozen sections. The paraffin-embedded spinal cord segment was sectioned longitudinally into 4 μm thick slices. The paraffin sections were deparaffinized and rehydrated in PBS. Hematoxylin and eosin (H&E) and cresyl violet were used for the H&E and Nissl staining respectively. The frozen sections were dried and separately with Prussian blue and DAB staining.

### Transmission electron microscopy

Cell samples were fixed in 2.5% glutaraldehyde for 2 h at room temperature and then stored at 4 °C overnight. Subsequently, samples were post-fixed with 1% osmium tetroxide for 1 h, washed, dehydrated through an ethanol gradient (30, 50, 70 and 95%, 5 min per step), embedded and polymerized at 60 °C for 48 h. Ultrathin sections of 85 nm were cut and observed in a Tecnai 12 BioTwin transmission electron microscope (FEI Company, Eindhoven, The Netherlands) at 120 keV.

For tissue microscopy, the T10 segment of spinal cord was isolated and sliced in 1 mm thick longitudinal sections. The fixed tissues were embedded using EPON as previously described. Ultrathin sections of 70 nm were cut using an ultramicrotome (Leica Microsystems, UC6) with a diamond knife (Diatome, Biel, Switzerland) and stained with 1.5% uranyl acetate at 37 °C for 15 min and lead citrate solution for 4 min. Electron micrographs were taken with a JEM-2100 Plus Transmission Electron Microscope (JEOL), equipped with Camera OneView 4 K 16 bit (Gatan) and software DigitalMicrograph (Gatan).

For analysis, the length and morphology of each mitochondrion was determined in Image J software following manual drawing of single organelle. All parameters obtained from one field of view were averaged together.

### BMS (basso mouse scale) score

Locomotor function of mice was evaluated using an open-field score, the Basso Mouse Scale (BMS) as previously reported^19^. Briefly, on the 0, 1st, 3rd, 7th, 14th, 21st, 28th, 35th, 42nd days after injury, all mice were allowed to crawl freely and evaluated by two independent researchers who were blind to the treatment groups. The score was based on the ankle movement, plantar placing, plantar stepping and motor coordination.

### Footprint test

Footprint test was used to observe the gait recovery and motor coordination according to our established previous article^19^. Briefly, on the 42nd day after SCI, mice were encouraged to crawl along a straight line on a piece of white paper three times with their left and right hind paws painted by blue and red dye, respectively. Footprint traces were printed on the paper and digitized by the camera.

### Motor evoked potential (MEP) detection

The function of motor nervous system of lower limbs was tested by MEP detection on the 42nd day after the SCI injury. BL-420A/F Data Acquisition Analysis System (TECHMAN SOFT) was applied. The motor cortex of mice was exposed and touched by stimulating electrodes. The recording electrodes were placed on the contralateral sciatic nerve. The latency and amplitude of the first evoked peak were selected as parameters for assessment of the function of the motor neuron.

### Measurements of ROS and MDA

Intracellular ROS levels were determined by DHE Assay Kit (ab236206, Abcam) according to the manufacturer's instruction. Briefly, cells were incubated in DMEM containing 10 µmol/L DHE at 37 °C for 30 min in the dark. After washing, labeled cells were evaluated by flow cytometry. Data were expressed as the MFI and analyzed by FlowJo v10.6.2 software.

MDA was used for the evaluation of oxidative stress. MDA assay kit (S0131S, Beyotime) was applied to this measurement and the procedures followed the manufacturer's protocol. The protein extraction of cell samples and standard solution of MDA were prepared and respectively incubated with MDA working solutions at 100 °C for 15 min, followed by centrifugation at 1000 g for 10 min. The absorbance of the supernatant at 532 nm was measured by the microplate reader (Sunrise, TECAN), representing the relative concentration of protein extraction.

### Measurements of intracellular total iron

The level of intracellular total iron was determined using Intracellular Iron Colorimetric Assay Kit (E1042, APPLYGEN) according to the instruction. After processed with working solution at 60°C for 15 min, cell lysate and standard sample were incubated with iron probe at 37 °C for 30 min. The absorbance at 550 nm was measured by the microplate reader (Sunrise, TECAN). Concentrations of intracellular total iron were calculated according to standard curve. Protein concentrations of each sample should be determined for the measurement of intracellular total iron per unit of protein.

### RNA-seq processing and data analysis

HT22 cells were divided into oeNC-RSL3 (transfected with empty vector lentivirus) and oeCLU-RSL3 (transfected with clusterin-overexpressing lentivirus) groups, following ferroptosis induction with 1 μM RSL3 for 12 h, total RNA was extracted using TRIzol reagent. RNA quality was verified by NanoDrop 2000 (A260/A280 > 1.8) and Agilent 2100 Bioanalyzer (RIN ≥ 7.0). Qualified samples underwent library preparation with the Illumina TruSeq Stranded mRNA LT Kit and were sequenced on Illumina Novaseq 6000 by Haplox Biotechnology (Jiangxi, China). Raw data were quality-controlled (FastQC), trimmed (Trimmomatic), and aligned to the mouse GRCm39 genome (HISAT2). Gene expression quantification was performed using featureCounts. Differential expression analysis was conducted with DESeq2 (thresholds: |log₂FC| > 1 and FDR < 0.05), with results visualized in volcano plots. Significantly differentially expressed genes underwent functional enrichment via GO analysis (BP/MF/CC categories, ClusterProfiler) and KEGG pathway analysis (KOBAS-i, FDR < 0.05). Three biological replicates per group ensured statistical reliability.

### Statistics and reproducibility

All experiments were carried out with at least three biological replicates and successful reproducibility was shown. Data are reported as the mean ± standard deviation (SD) of at least three independent experiments. Sample sizes are all presented in the figure legends. Statistical analysis between two groups was performed using unpaired t-test. Statistical analysis between multiple groups was performed by one-way ANOVA with Tukey's or Dunnett's multiple comparison test, or Two-way ANOVA with Tukey's, Dunnett's or Sidák's multiple comparisons test. All data were analyzed using GraphPad Software. A two-sided p-value < 0.05 was considered to be statistically significant. The level of significance defined as p < 0.05 (*), p < 0.01 (**), p < 0.001 (***). Besides, ns indicates not significant difference as p > 0.05.

## Results

### Expression of neuronal CLU after SCI is associated with neuronal ferroptosis

**Figure 1:**
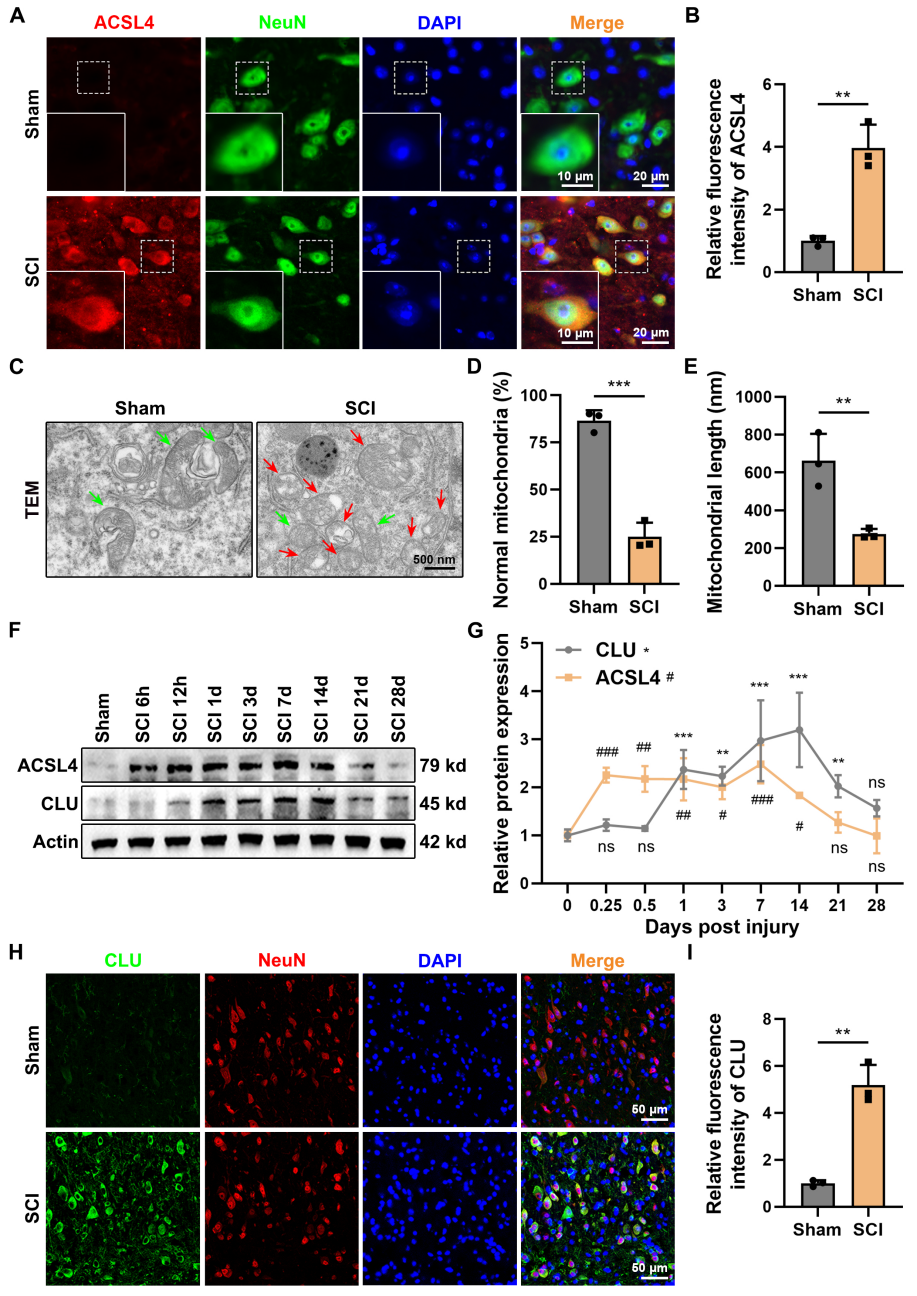
** Expression of neuronal CLU after spinal cord injury is associated with neuronal ferroptosis. (A)** Representative immunofluorescent staining images of the ferroptosis marker ACSL4 in the spinal cord lesions at 1 dpi of mice from Sham or Spinal Cord Injury (SCI) group. Neurons were marked by NeuN. Scale bar, 20 μm for original and 10 μm for enlarged pictures. **(B)** Quantification of the mean fluorescence intensity of ACSL4 in** (A)**. (n = 3 biological repeats for each group; Unpaired t test). **(C)** Representative transmission electron microscope (TEM) images of neurons at 1 dpi of mice from Sham or SCI group *in vivo*. Red and green arrows indicate damaged and normal mitochondria, respectively. Scale bar, 500 nm. **(D)** Quantification of the percentage of mitochondria with normal morphology in **(C)**. (n = 3 biological repeats for each group; Unpaired t test). **(E)** Quantification of the average mitochondrial length (nanometer, nm) in** (C)**. (n = 3 biological repeats for each group; Unpaired t test). **(F-G)** Western blot analysis and quantification of relative temporal changes in CLU and ACSL4 protein expression levels after SCI, using spinal cord tissue from the injury lesions of mice at different time points post-injury (6 hours, 12 hours, 1 day, 3 days, 7 days, 14 days, 21 days, and 28 days). (n = 3 biological repeats for each group; Two-way ANOVA with Dunnett's multiple comparisons test). **(H)** Representative immunofluorescent staining images of CLU protein in the spinal cord lesions at 1 dpi of mice from Sham or SCI group. Neurons were marked by NeuN. Scale bar, 50 μm. **(I)** Quantification of the mean fluorescence intensity of CLU in **(H)**. (n = 3 biological repeats for each group; Unpaired t test). Two-sided comparison; All data are mean ± SD; Error bars represent SDs; *p < 0.05, **p < 0.01, ***p < 0.001, ns, not significant, p > 0.05.

Spinal cord injury (SCI) induces a complex pathological cascade, among which neuronal ferroptosis has been identified as a crucial contributor to secondary injury. Immunofluorescence staining showed that ACSL4 expression in the spinal cord tissue was markedly increased in the SCI group compared with the sham group **(Figures 1A-1B)**. Transmission electron microscopy (TEM) revealed shrunken mitochondria with reduced or vanished cristae and ruptured outer membranes **(Figures 1C-1E)**, confirming the presence of neuronal ferroptosis after SCI.

Western blot (WB) analysis demonstrated that CLU expression gradually increased after SCI, becoming significantly upregulated from day 1 and remaining elevated up to day 21 **(Figures 1F-1G)**. ACSL4 displayed a similar temporal trend, peaking between days 1 and 7 and then declining after day 14. Immunofluorescence double staining showed that CLU was primarily localized in the neuronal cytoplasm and its expression was markedly enhanced after injury** (Figures 1H-1I)**. This upregulation is consistent with CLU's role as a stress-responsive protein and likely represents an intrinsic adaptive mechanism whereby neurons attempt to mitigate injury^25^,^31^. However, the substantial neuronal loss observed despite this increase suggests that the endogenous compensatory response is insufficient to confer sustained protection against the progression of damage.

These results collectively indicate that neuronal CLU expression is upregulated following SCI and is closely associated with neuronal ferroptosis.

### Exogenous addition of CLU protein inhibits neuronal ferroptosis

**Figure 2:**
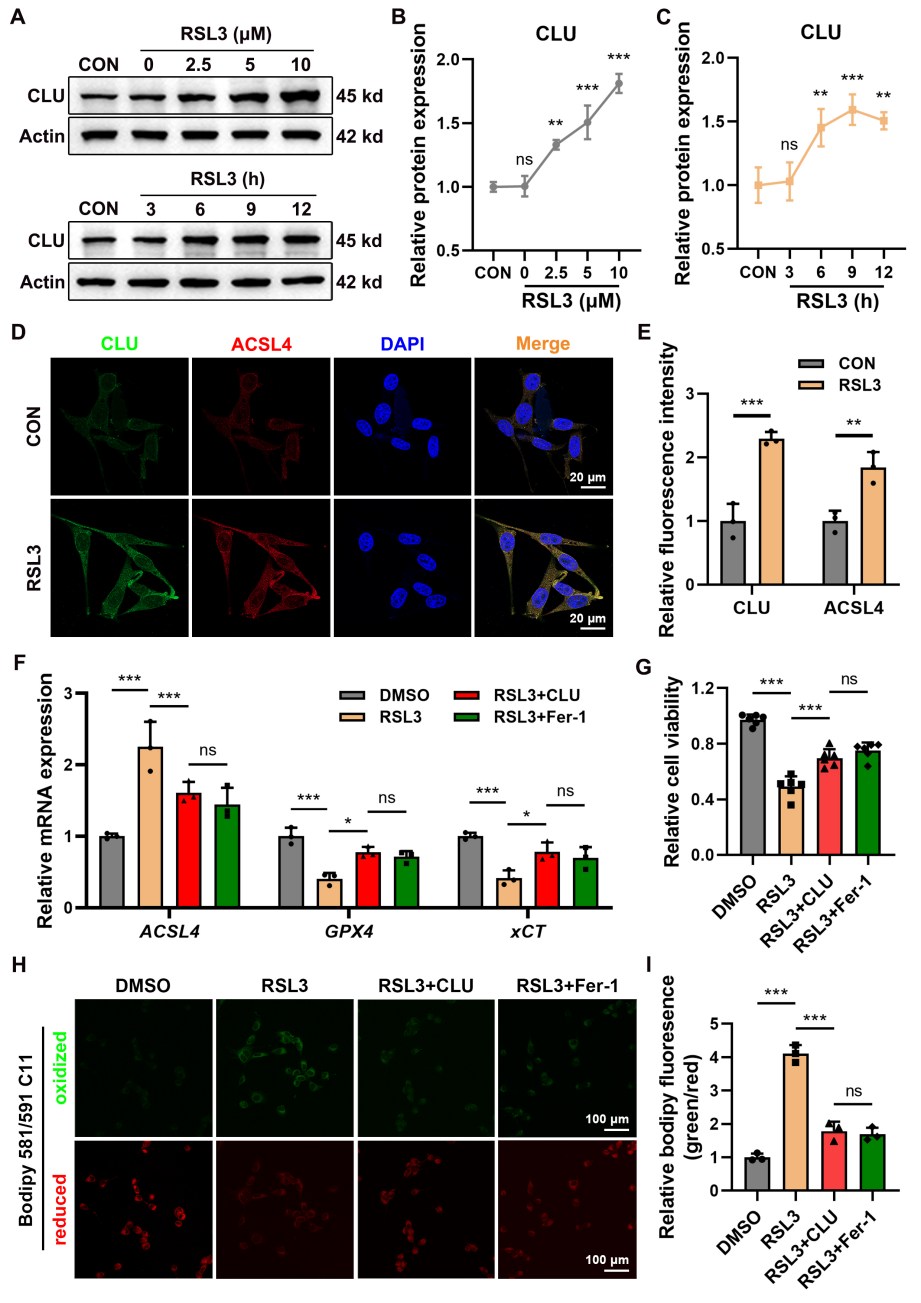
** Exogenous addition of CLU protein inhibits neuronal ferroptosis. (A)** Western blot analysis of the CLU protein expression in neuronal cell line (HT22) treated with different concentrations (0, 2.5, 5, 10 μM) of RSL3 or different durations (3, 6, 9, 12 h) with 5 μM RSL3. **(B)** Quantification of relative CLU protein expression levels under different RSL3 concentrations in** (A)**. (n=3 biological repeats for each group; One-way ANOVA with Dunnett's multiple comparisons test). **(C)** Quantification of relative CLU protein expression levels under different RSL3 treatment durations in** (A)**. (n=3 biological repeats for each group; One-way ANOVA with Dunnett's multiple comparisons test). **(D)** Representative immunofluorescent staining images showing co-localization of CLU and ACSL4 in HT22 cells. CLU is labeled in green and ACSL4 in red. HT22 cells were stimulated with 5 μM RSL3 to induce ferroptosis. Scale bar, 20 μm. **(E)** Quantification of fluorescence intensity of CLU and ACSL4 in **(D)**. (n = 3 biological repeats for each group; Two-way ANOVA with Sidák's multiple comparisons test). **(F)** qPCR analysis of relative mRNA expression of ferroptosis-related genes, including *ACSL4*, *GPX4* and *xCT* in HT22 cells treated with: DMSO, RSL3 alone, RSL3 plus recombinant CLU protein, or RSL3 plus ferroptosis inhibitor Fer-1 (ferroptosis inhibitor). (n = 3 biological repeats for each group; Two-way ANOVA with Tukey's multiple comparisons test). **(G)** Quantification of cell viability in HT22 treated with: DMSO, RSL3 alone, RSL3 plus recombinant CLU protein, or RSL3 plus Fer-1. (n=3 biological repeats for each group; One-way ANOVA with Tukey's multiple comparisons test). **(H)** Representative fluorescence staining images showing lipid peroxidation levels in HT22 cells under different treatments (DMSO, RSL3, RSL3+CLU, RSL3+Fer-1). Green represents oxidized state and red represents reduced state. Scale bar, 100 μm. **(I)** Quantification of relative lipid peroxidation levels calculated by bodipy fluorescence intensity (oxidized state / reduced state) in **(H)**. (n=3 biological repeats for each group; One-way ANOVA with Tukey's multiple comparisons test). Two-sided comparison; All data are mean ± SD; Error bars represent SDs; *p < 0.05, **p < 0.01, ***p < 0.001, ns, not significant, p > 0.05. See also **Supplementary Figure 1**.

To deeply investigate CLU expression and potential function during neuronal ferroptosis, we treated HT22 cells with the ferroptosis inducer RSL3. WB analysis revealed that CLU protein expression was upregulated in a dose- and time-dependent manner following RSL3 stimulation **(Figures 2A-2C)**. This inducible expression pattern is consistent with the CLU dynamics observed in our SCI mouse model, suggesting a universal neuronal response to ferroptotic stress.

Given that the subcellular localization of CLU is closely linked to its function (cytoplasmic CLU is often associated with protective mechanisms, while nuclear CLU may be pro-death)^32^-^34^, we next we used immunofluorescent staining to study its subcellular distribution. The results of confocal microscopy showed that under ferroptotic conditions, CLU was primarily upregulated in the cytoplasm. Notably, this cytoplasmic CLU accumulation showed a high degree of synchronization and co-localization with the key ferroptosis driver ACSL4 **(Figures 2D-2E)**, strongly suggesting a close functional interaction between the two proteins in regulating neuronal ferroptosis.

To determine whether CLU can directly inhibit ferroptosis, we examined its effect on key ferroptotic markers and processes. Treating HT22 cells with recombinant CLU protein (rCLU) effectively counteracted the transcriptional changes induced by RSL3. Specifically, rCLU rescued the suppression of the anti-ferroptotic genes *xCT* and *GPX4*^17^ and attenuated the upregulation of the pro-ferroptotic gene *ACSL4*^35^, with the latter confirmed at the protein level by immunofluorescence **(Figures 2F and S2A-S2C)**. This transcriptional reprogramming translated into functional protection, as rCLU pretreatment significantly restored cell viability after RSL3 challenge, to an extent comparable to the ferroptosis inhibitor Ferrostatin-1 (Fer-1) **(Figure 2G)**. Mechanistically, the protective effect was attributable to the suppression of lipid peroxidation, a hallmark of ferroptosis^36^. rCLU potently inhibited RSL3-induced lipid peroxidation, as measured by BODIPY C11 staining, with efficacy similar to Fer-1 **(Figure 2H-2I)**.

Collectively, these results demonstrate that exogenous CLU protein acts as an endogenous ferroptosis inhibitor by mimicking endogenous neuroprotective effect.

### Endogenous overexpression of CLU inhibits neuronal ferroptosis

**Figure 3:**
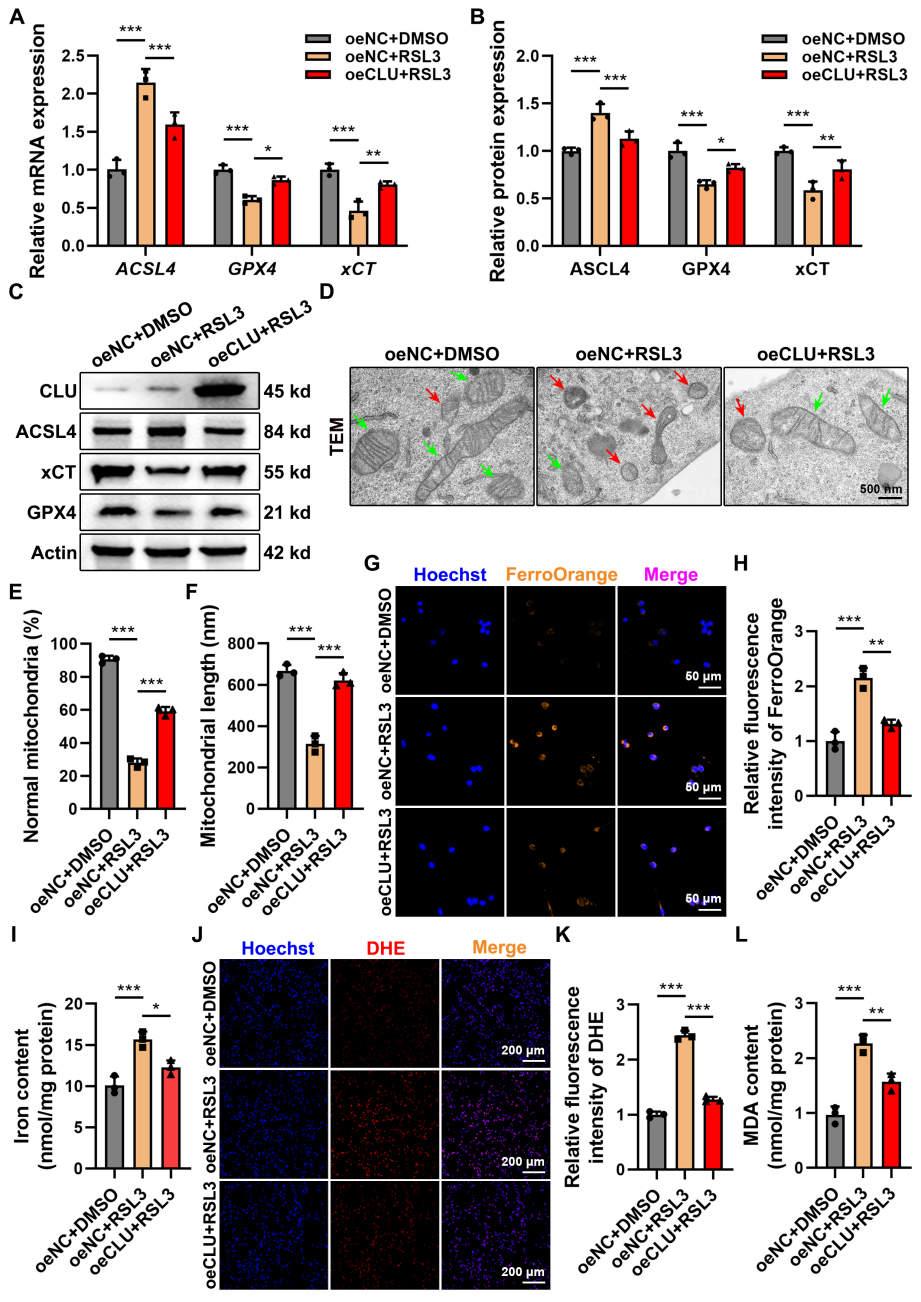
** Endogenous overexpression of CLU *in vitro* inhibits neuronal ferroptosis. (A)** qPCR analysis of relative mRNA expression of ferroptosis-related genes, including *ACSL4*, *GPX4*, *xCT*, in HT22 cells with different treatment: oeNC + DMSO, oeNC + RSL3, and oeCLU + RSL3. (n = 3 biological repeats for each group; Two-way ANOVA with Tukey's multiple comparisons test). **(B)** Quantification of relative protein expression levels in **(C)**. (n = 3 biological repeats for each group; Two-way ANOVA with Tukey's multiple comparisons test). **(C)** Western blot analysis of ferroptosis-related proteins, including ACSL4, GPX4, xCT, in HT22 cells with different treatment: oeNC + DMSO, oeNC + RSL3, and oeCLU + RSL3. **(D)** Representative TEM images of HT22 cells with different treatment: oeNC + DMSO, oeNC + RSL3, and oeCLU + RSL3 *in vitro*. Red and green arrows indicate damaged and normal mitochondria, respectively. Scale bar, 500 nm. **(E)** Quantification of the percentage of mitochondria with normal morphology in** (D)**. (n=3 biological repeats for each group; One-way ANOVA with Tukey's multiple comparisons test). **(F)** Quantification of the average mitochondrial length (nm) in **(D)**. (n=3 biological repeats for each group; One-way ANOVA with Tukey's multiple comparisons test). **(G)** Representative immunofluorescent staining images of neurons of HT22 cells with different treatment: oeNC + DMSO, oeNC + RSL3, and oeCLU + RSL3 *in vitro*. FerroOrange (orange-yellow), a ferrous ion (Fe²⁺) probe, was used to detect intracellular ferrous iron accumulation. Scale bar, 50 μm. **(H)** Quantification of FerroOrange fluorescence intensity in **(G)**. (n=3 biological repeats for each group; One-way ANOVA with Tukey's multiple comparisons test). **(I)** Quantification of total iron ion levels in HT22 cells with different treatment: oeNC + DMSO, oeNC + RSL3, and oeCLU + RSL3 *in vitro*. (n=3 biological repeats for each group; One-way ANOVA with Tukey's multiple comparisons test). **(J)** Representative immunofluorescent staining images reflecting intracellular superoxide anion levels in HT22 cells with different treatment: oeNC + DMSO, oeNC + RSL3, and oeCLU + RSL3 *in vitro*. DHE reacts with intracellular superoxide ions to produce ethidium bromide, which then binds to RNA and DNA, emitting red fluorescence. The intensity of red fluorescence is proportional to the level of intracellular superoxide anions. Scale bar, 200 μm. **(K)** Quantification of DHE fluorescence intensity in** (J)**. (n=3 biological repeats for each group; One-way ANOVA with Tukey's multiple comparisons test). **(L)** Quantification of intracellular MDA levels in HT22 cells with different treatment: oeNC + DMSO, oeNC + RSL3, and oeCLU + RSL3 *in vitro*. (n=3 biological repeats for each group; One-way ANOVA with Tukey's multiple comparisons test). Two-sided comparison; All data are mean ± SD; Error bars represent SDs; *p < 0.05, **p < 0.01, ***p < 0.001. See also **Supplementary Figure 2**.

To investigate CLU's role in ferroptosis, we established stable CLU-overexpressing neuronal lines (oeCLU)** (Figures S2A-S2C)**. Upon RSL3 treatment, CLU overexpression reversed key ferroptosis-related transcriptional changes: it elevated mRNA levels of the anti-ferroptotic genes *GPX4* and *xCT*, while reducing expression of the pro-ferroptotic gene *ACSL4*, as confirmed at the protein level by WB** (Figures 3A-3C)**. Immunofluorescence further showed that RSL3-induced ACSL4 intensity was markedly reduced in oeCLU cells **(Figures S2D-S2F)**.

Functionally, TEM revealed that CLU overexpression protected mitochondria from ferroptotic damage, preserving their structure, cristae, and membrane integrity under RSL3 stimulation, with increased mitochondrial number and length compared to controls** (Figures 3D-3F)**.

Mechanistically, CLU also attenuated iron accumulation, a hallmark of ferroptosis. FerroOrange staining and total iron assays showed significantly lower Fe²⁺ and total iron levels in RSL3-treated oeCLU cells** (Figures 3G-3I)**. Moreover, CLU reduced RSL3-induced superoxide anions (detected by DHE staining) and malondialdehyde (MDA), indicating suppression of lipid peroxidation **(Figures 3J-3L)**.

Together, these results demonstrate that CLU overexpression inhibits neuronal ferroptosis by modulating ferroptosis-related gene expression, preserving mitochondrial integrity, limiting iron accumulation, and reducing oxidative stress and lipid peroxidation.

### Endogenous knockdown of CLU aggravates neuronal ferroptosis

**Figure 4:**
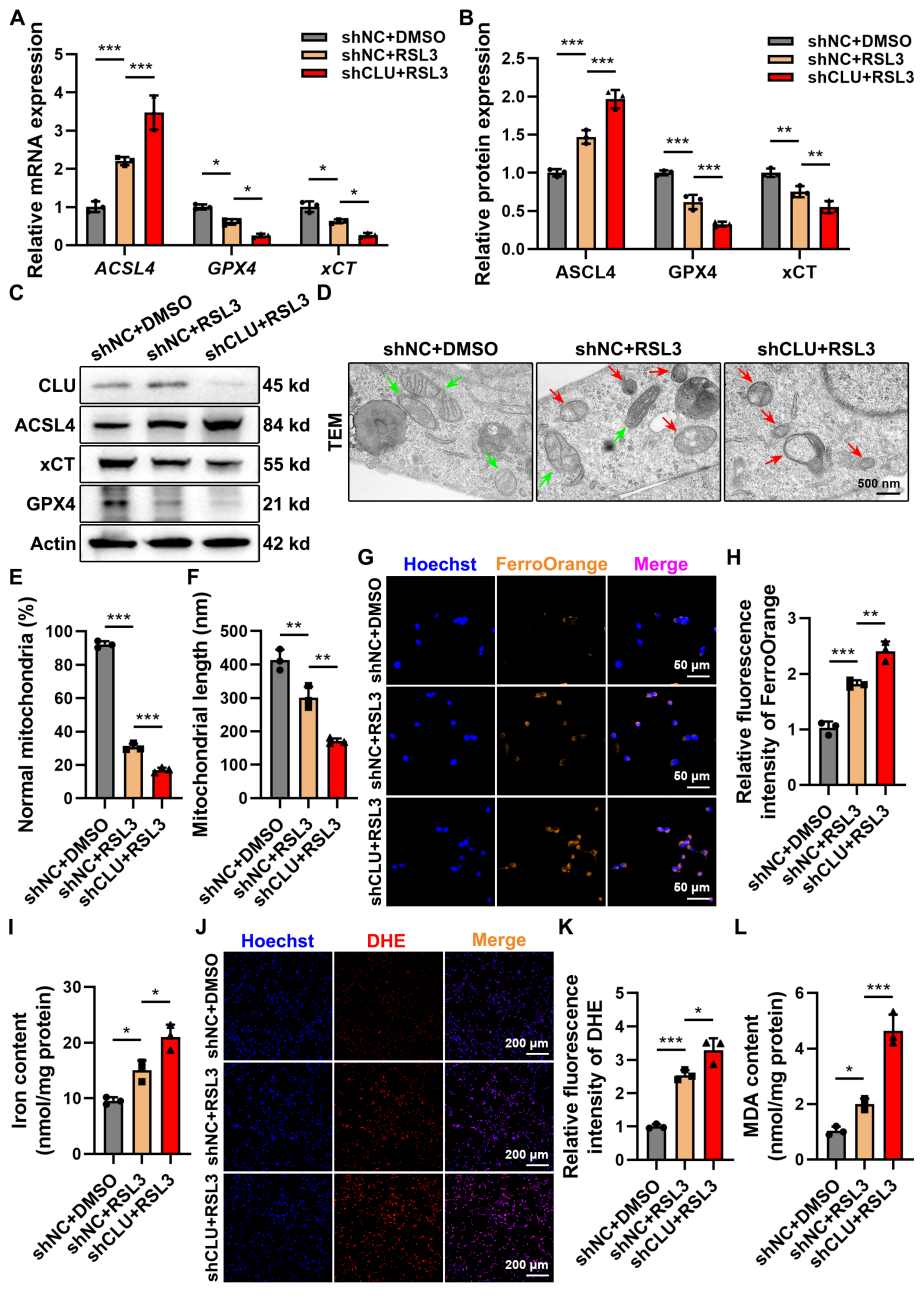
** Knockdown of CLU *in vitro* aggravates neuronal ferroptosis. (A)** qPCR analysis of relative mRNA expression of ferroptosis-related genes, including *ACSL4*, *GPX4*, *xCT*, in HT22 cells with different treatment: shNC + DMSO, shNC + RSL3, and shCLU + RSL3. (n = 3 biological repeats for each group; Two-way ANOVA with Tukey's multiple comparisons test). **(B)** Quantification of relative protein expression levels in **(C)**. (n = 3 biological repeats for each group; Two-way ANOVA with Tukey's multiple comparisons test). **(C)** Western blot analysis of ferroptosis-related proteins, including ACSL4, GPX4, xCT, in HT22 cells with different treatment: shNC + DMSO, shNC + RSL3, and shCLU + RSL3. **(D)** Representative TEM images of HT22 cells with different treatment: shNC + DMSO, shNC + RSL3, and shCLU + RSL3 *in vitro*. Red and green arrows indicate damaged and normal mitochondria, respectively. Scale bar, 500 nm. **(E)** Quantification of the percentage of mitochondria with normal morphology in** (D)**. (n=3 biological repeats for each group; One-way ANOVA with Tukey's multiple comparisons test). **(F)** Quantification of the average mitochondrial length (nm) in **(D)**. (n=3 biological repeats for each group; One-way ANOVA with Tukey's multiple comparisons test). **(G)** Representative immunofluorescent staining images of neurons of HT22 cells with different treatment: shNC + DMSO, shNC + RSL3, and shCLU + RSL3 *in vitro*. FerroOrange (orange-yellow), a ferrous ion (Fe²⁺) probe, was used to detect intracellular ferrous iron accumulation. Scale bar, 50 μm. **(H)** Quantification of FerroOrange fluorescence intensity in **(G)**. (n=3 biological repeats for each group; One-way ANOVA with Tukey's multiple comparisons test). **(I)** Quantification of total iron ion levels in HT22 cells with different treatment: shNC + DMSO, shNC + RSL3, and shCLU + RSL3 *in vitro*. (n=3 biological repeats for each group; One-way ANOVA with Tukey's multiple comparisons test). **(J)** Representative immunofluorescent staining images reflecting intracellular superoxide anion levels in HT22 cells with different treatment: shNC + DMSO, shNC + RSL3, and shCLU + RSL3 *in vitro*. DHE reacts with intracellular superoxide ions to produce ethidium bromide, which then binds to RNA and DNA, emitting red fluorescence. The intensity of red fluorescence is proportional to the level of intracellular superoxide anions. Scale bar, 200 μm. **(K)** Quantification of DHE fluorescence intensity in** (J)**. (n=3 biological repeats for each group; One-way ANOVA with Tukey's multiple comparisons test). **(L)** Quantification of intracellular MDA levels in HT22 cells with different treatment: shNC + DMSO, shNC + RSL3, and shCLU + RSL3 *in vitro*. (n=3 biological repeats for each group; One-way ANOVA with Tukey's multiple comparisons test). Two-sided comparison; All data are mean ± SD; Error bars represent SDs; *p < 0.05, **p < 0.01, ***p < 0.001. See also **Supplementary Figure 3**.

To further validate the cytoprotective role of CLU, we established stable lentiviral CLU-knockdown (shCLU) neuronal cell lines **(Figures S3A-S3C)**. Suppression of CLU expression markedly intensified ferroptotic injury, as evidenced by upregulation of ACSL4 and concomitant downregulation of GPX4 and xCT, thereby enhancing cellular sensitivity to RSL3 **(Figures 4A-4C)**. Immunofluorescence confirmed these findings, showing a pronounced increase in ACSL4 fluorescence intensity following RSL3 exposure in shCLU cells **(Figures S3D-S3F)**.

TEM revealed typical ferroptotic mitochondrial damage in RSL3-treated shNC cells, which became more pronounced in shCLU+RSL3 cells, indicating that CLU deficiency abolishes its protective effect against ferroptotic mitochondrial injury **(Figures 4D-4F)**. Consistently, FerroOrange staining demonstrated a significant elevation in both Fe²⁺ and total iron accumulation in shCLU+RSL3 cells compared with controls **(Figures 4G-4I)**. Moreover, DHE staining and MDA quantification showed that CLU knockdown led to a marked increase in superoxide (O₂⁻˙) generation and lipid peroxidation levels **(Figures 4J-4L)**, further supporting the loss of CLU-mediated antioxidant protection.

Collectively, these data identify CLU as a pivotal endogenous regulator of neuronal ferroptosis. Its expression determines neuronal vulnerability to ferroptotic stress: overexpression confers substantial protection, whereas knockdown exacerbates ferroptotic damage. These findings provide a mechanistic foundation for targeting CLU as a potential therapeutic strategy in spinal cord injury.

### CLU inhibits neuronal ferroptosis by activating the PI3K-AKT-mTOR pathway

**Figure 5:**
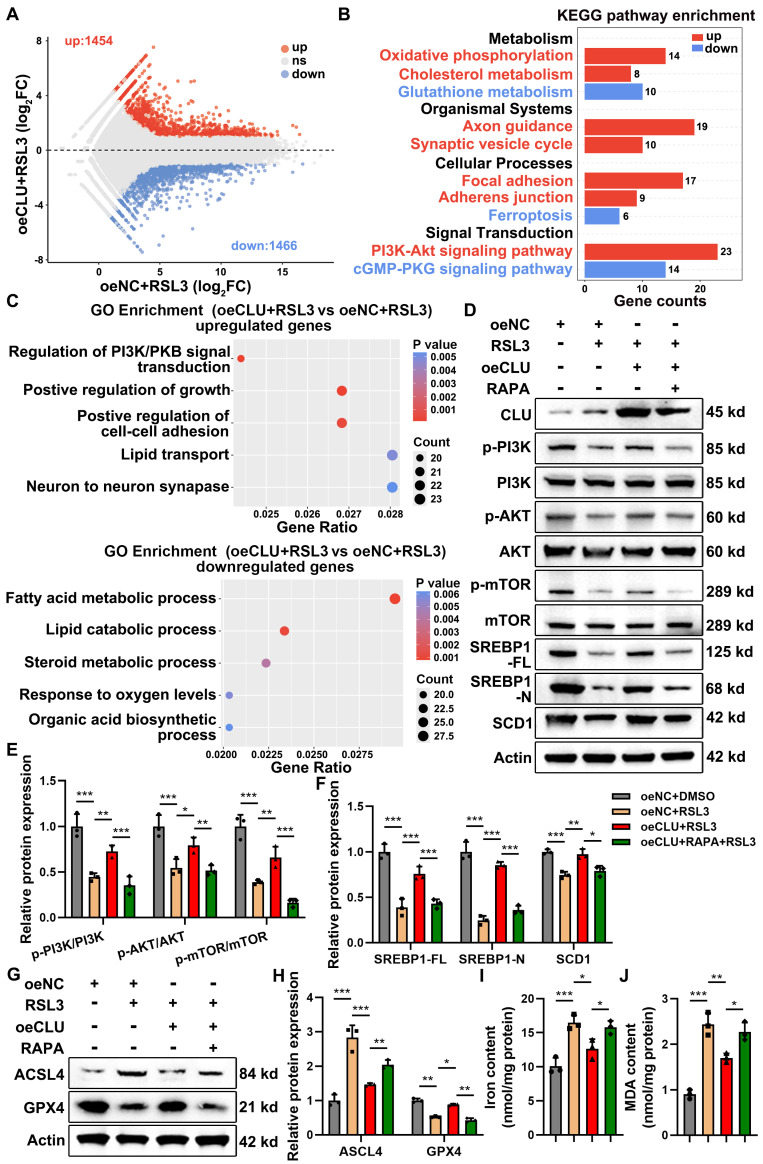
** CLU inhibits neuronal ferroptosis by activating the PI3K-AKT-mTOR pathway *in vitro*. (A)** Scatter plot (Volcano plot) of differentially expressed genes (DEGs), showing gene expression changes between the oeNC+RSL3 group and the oeCLU+RSL3 group. Red dots represent significantly upregulated genes (up: 1454 genes), blue dots represent significantly downregulated genes (down: 1466 genes), and grey dots represent genes with not significant difference (ns). Used for preliminary screening of genes with significant expression differences between the two groups. **(B)** Bar chart of Kyoto Encyclopedia of Genes and Genomes (KEGG) pathway enrichment analysis, showing the enrichment of DEGs in different KEGG pathways. The x-axis represents the gene count enriched in the pathway, the y-axis represents the pathway categories and names. Red represents pathways enriched with upregulated genes, and blue represents pathways enriched with downregulated genes in the comparison between the oeCLU+RSL3 group and the oeNC+RSL3 group. **(C)** Gene Ontology (GO) enrichment analysis results, showing the functional enrichment of upregulated genes (upregulated genes) and downregulated genes (downregulated genes) in the comparison between the oeCLU+RSL3 group and the oeNC+RSL3 group. **(D)** Western blot analysis of PI3K-AKT-mTOR signaling pathway-related proteins and lipid metabolism-related proteins, including SREBP1 and SCD1, in HT22 with different treatment groups: oeNC (empty vector), oeNC+RSL3 (ferroptosis inducer), oeCLU (CLU overexpression) +RSL3, oeCLU+RSL3+RAPA (mTOR inhibitor). **(E)** Quantification of relative pathway protein phosphorylation levels, calculating the relative protein expression of p-PI3K/PI3K, p-AKT/AKT, p-mTOR/mTOR in** (D)**. (n = 3 biological repeats for each group; Two-way ANOVA with Tukey's multiple comparisons test). **(F)** Quantification of relative lipid metabolism-related protein expression levels, including the full-length (FL) and nuclear (N) form of SREBP1 and SCD1, in** (D)**. (n = 3 biological repeats for each group; Two-way ANOVA with Tukey's multiple comparisons test). **(G)** Western blot analysis of ferroptosis-related proteins, including GPX4 and ACSL4, in HT22 with different treatment groups: oeNC, oeNC+RSL3, oeCLU+RSL3, oeCLU+RSL3+RAPA. **(H)** Quantification of relative ferroptosis-related protein expression levels, including ACSL4 and GPX4 in** (G)**. (n = 3 biological repeats for each group; Two-way ANOVA with Tukey's multiple comparisons test). **(I)** Quantification of intracellular total iron ion levels in HT22 with different treatment groups: oeNC, oeNC+RSL3, oeCLU+RSL3, oeCLU+RSL3+RAPA. (n=3 biological repeats for each group; One-way ANOVA with Tukey's multiple comparisons test). **(J)** Quantification of intracellular MDA levels, reflecting the regulation of cellular lipid peroxidation levels by the treatment factors in HT22 with different treatment groups: oeNC, oeNC+RSL3, oeCLU+RSL3, oeCLU+RSL3+RAPA. (n=3 biological repeats for each group; One-way ANOVA with Tukey's multiple comparisons test). Two-sided comparison; All data are mean ± SD; Error bars represent SDs; *p < 0.05, **p < 0.01, ***p < 0.001. See also **Supplementary Figure 4**.

To elucidate the underlying mechanism, we systematically profiled downstream signaling events. Transcriptomic and pathway enrichment analyses identified the PI3K-AKT-mTOR pathway as the most significantly enriched in CLU-overexpressing neurons under ferroptotic stress** (Figures 5A-5C)**. Corroborating these findings, CLU overexpression in RSL3-treated HT22 cells markedly rescued the reduced phosphorylation of PI3K, AKT, and mTOR **(Figures 5D-5E)**. This CLU-mediated activation was entirely dependent on the pathway, as it was abolished by the mTOR inhibitor rapamycin (RAPA) **(Figures 5D-5E)**, establishing that CLU inhibits neuronal ferroptosis primarily via PI3K-AKT-mTOR signaling.

Next, we further interrogated the functional consequences of pathway inhibition. Treatment with rapamycin in CLU-overexpressing cells abrogated the ferroptosis-resistant phenotype, which was evident from the increased ACSL4 expression, decreased GPX4 levels **(Figures 5G-5H and S4A-S4C)**, and the concomitant rise in iron accumulation and MDA production **(Figures 5I-5J)**.

To delineate the downstream transcriptional mechanism linking mTOR activation to ferroptosis suppression, we investigated the lipogenic regulator SREBP1 and its target SCD1, a known ferroptosis suppressor via monounsaturated fatty acids (MUFAs) synthesis^37^. The protein levels of full-length SREBP1 (SREBP1-FL), its transcriptionally active nuclear form (SREBP1-N), and SCD1 all correlated with mTOR activity. CLU overexpression upregulated these factors, and this induction was blunted by rapamycin** (Figures 5D and 5F)**. Thus, CLU activates mTOR to promote the proteolytic maturation of SREBP1 and the subsequent expression of SCD1, thereby conferring ferroptosis resistance.

### Overexpression of CLU *in vivo* inhibits neuronal ferroptosis by activating PI3K-AKT- mTOR pathway

**Figure 6:**
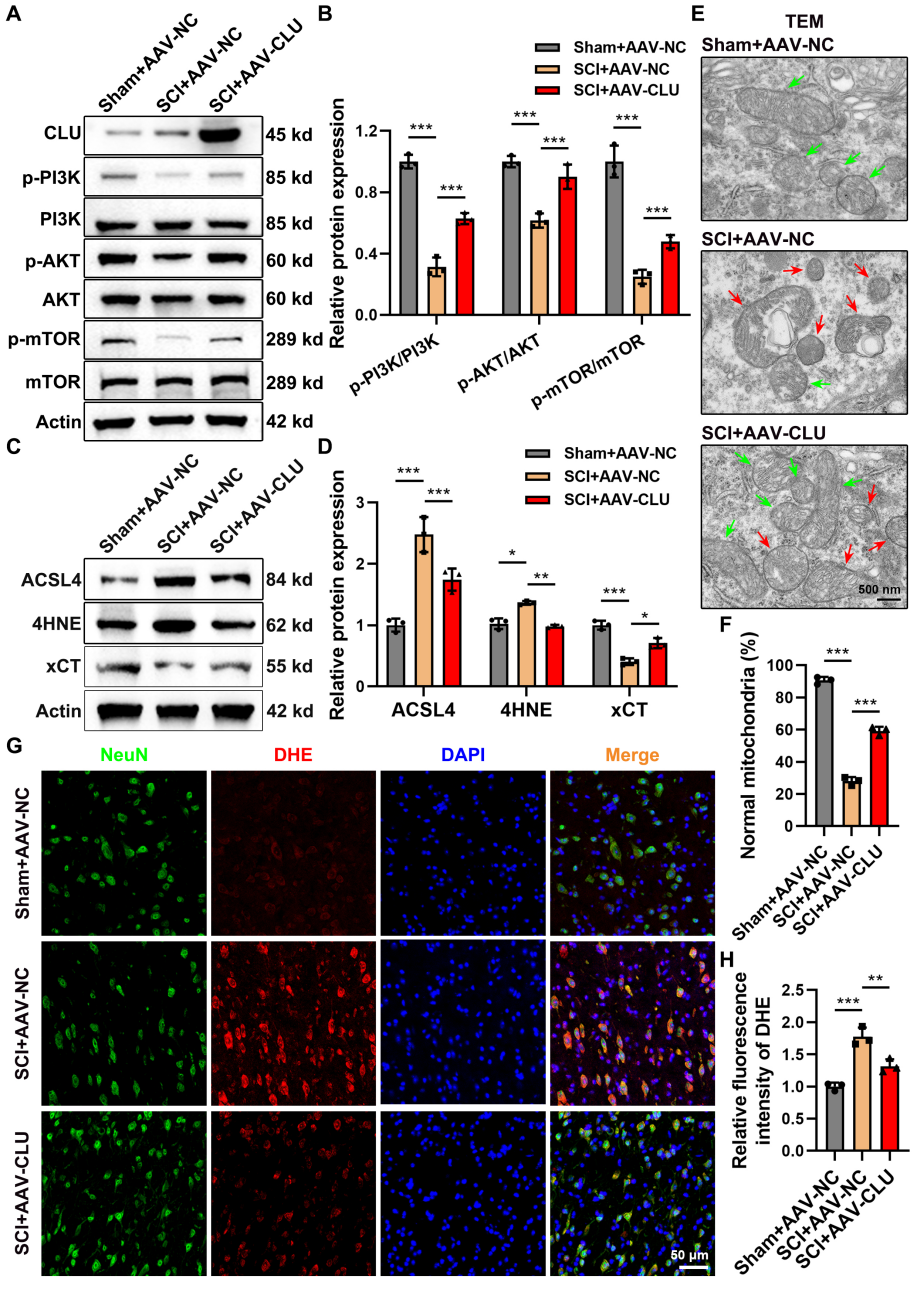
** Overexpression of CLU *in vivo* inhibits neuronal ferroptosis by activating the PI3K-AKT-mTOR pathway. (A)** Western blot analysis of PI3K-AKT-mTOR signaling pathway-related proteins in the spinal cord lesions at 7 dpi of mice from Sham+AAV-NC (Sham surgery + empty virus control), SCI+AAV-NC (SCI + empty virus) and SCI+AAV-CLU (SCI + CLU overexpression virus) group. **(B)** Quantification of relative pathway protein phosphorylation levels, calculating the relative protein expression of p-PI3K/PI3K, p-AKT/AKT, p-mTOR/mTOR in **(A)**. (n = 3 biological repeats for each group; Two-way ANOVA with Sidák's multiple comparisons test). **(C)** Western blot analysis of relative ferroptosis-related protein expression levels in the spinal cord lesions at 7 dpi of mice from Sham+AAV-NC, SCI+AAV-NC and SCI+AAV-CLU group. **(D)** Quantification of relative protein expression levels in **(C)**. (n = 3 biological repeats for each group; Two-way ANOVA with Sidák's multiple comparisons test). **(E)** Representative transmission electron microscope (TEM) images of neurons *in vivo* at 7 dpi of mice from Sham+AAV-NC, SCI+AAV-NC and SCI+AAV-CLU group. Red and green arrows indicate damaged and normal mitochondria, respectively. Scale bar, 500 nm. **(F)** Quantification of the percentage of mitochondria with normal morphology in** (E)**. (n=3 biological repeats for each group; One-way ANOVA with Tukey's multiple comparisons test).** (G)** Representative immunofluorescent staining showing the distribution of reactive oxygen species (ROS) in neurons in spinal cord lesions at 7 dpi of mice from Sham+AAV-NC, SCI+AAV-NC and SCI+AAV-CLU group. Green: NeuN (neuronal marker protein), labeling neurons; Red: DHE (ROS probe), reflecting ROS levels. Scale bar, 50 μm. **(H)** Quantification of DHE fluorescence intensity in **(G)**. (n=3 biological repeats for each group; One-way ANOVA with Tukey's multiple comparisons test). Two-sided comparison; All data are mean ± SD; Error bars represent SDs; *p < 0.05, **p < 0.01, ***p < 0.001. See also **Supplementary Figure 5**.

Having established the role of CLU in activating the PI3K-AKT-mTOR pathway to inhibit ferroptosis *in vitro*, we next sought to validate this mechanism *in vivo*. Neuronal CLU was overexpressed in mice via AAV delivery (AAV-CLU) **(Figures S5A-S5D)**. WB analysis of spinal cord tissues revealed that SCI suppressed the PI3K-AKT-mTOR pathway, an effect that was significantly reversed by CLU overexpression, as evidenced by restored phosphorylation levels of PI3K, AKT, and mTOR **(Figures 6A-6B)**.

Consistent with this pathway activation, CLU overexpression markedly alleviated ferroptosis pathology at 7 days post-SCI. It counteracted the SCI-induced dysregulation of key ferroptotic proteins, elevating the anti-ferroptotic factor xCT while suppressing the pro-ferroptotic drivers ACSL4 and 4HNE **(Figures 6C-6D)**. The suppression of ACSL4 was visually confirmed by immunofluorescence** (Figures S5F-S5G)**. Ultrastructurally, TEM demonstrated that CLU preserved mitochondrial integrity, preventing the SCI-induced shrinkage, cristae loss, and outer membrane rupture **(Figures 6E-6F and S5E)**. Furthermore, CLU overexpression attenuated the core biochemical events of ferroptosis, reducing iron deposition, lipid peroxidation, and neuronal ROS levels **(Figures 6G-6H and S5H-S5J)**.

These results collectively demonstrate that CLU inhibits neuronal ferroptosis after SCI by activating the PI3K-AKT-mTOR pathway *in vivo*.

### Overexpression of CLU *in vivo* improves spinal cord injury

Building on the established mechanism that CLU inhibits ferroptosis via PI3K-AKT-mTOR activation, we next evaluated its integrated impact on neurological recovery and tissue repair following SCI.

**Figure 7:**
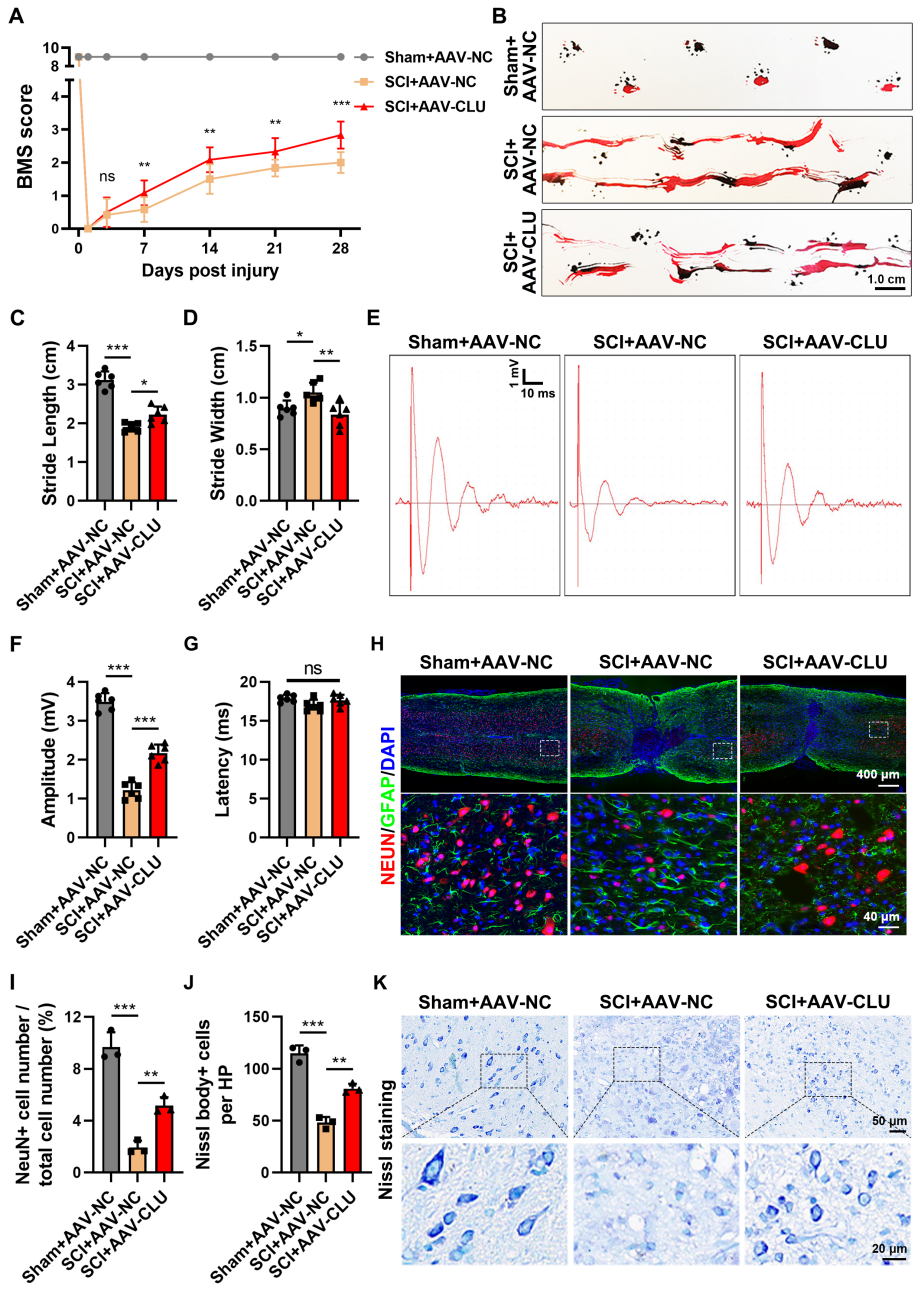
** Overexpression of CLU *in vivo* improves recovery of motor function following spinal cord injury. (A)** Measurement of Basso Mouse Scale (BMS) scores on day 0, 1, 3, 7, 14, 21, and 28 post injury (dpi) in mice from Sham+AAV-NC, SCI+AAV-NC and SCI+AAV-CLU group. (n = 6 biological repeats for each group; Two-way ANOVA with Tukey's multiple comparisons test). **(B)** Representative images of footprint test at 28 dpi of mice from Sham+AAV-NC, SCI+AAV-NC and SCI+AAV-CLU group. Scale bar, 1.0 cm. **(C-D)** Quantification of stride length and stride width for the mice in **(B)**. (n=6 biological repeats for each group; One-way ANOVA with Tukey's multiple comparisons test). **(E)** Representative diagrams of motor evoked potential (MEP) detection at 28 dpi of mice from Sham+AAV-NC, SCI+AAV-NC and SCI+AAV-CLU group. Scale bar, 1 mV and 10 ms. **(F-G)** Quantification of the amplitude of the first peak (mV) and the latency (ms) in **(E)**. (n=6 biological repeats for each group; One-way ANOVA with Tukey's multiple comparisons test). **(H)** Representative immunostaining pictures of the spinal cord at 28 dpi of mice from Sham+AAV-NC, SCI+AAV-NC and SCI+AAV-CLU group. Neurons were marked by NeuN and astrocytes were marked by GFAP to indicate the lesion area. Scale bar, 400 μm for original and 40 μm for enlarged pictures. **(I)** Quantification of the proportion of NeuN⁺ neurons in **(H)**, reflecting the relative survival proportion of neurons in spinal cord tissue. (n=3 biological repeats for each group; One-way ANOVA with Tukey's multiple comparisons test). **(J)** Quantification of the number of Nissl body⁺ cells per high-power field (HPF) in **(K)**. (n=3 biological repeats for each group; One-way ANOVA with Tukey's multiple comparisons test). **(K)** Representative images of Nissl staining at 28 dpi of mice from Sham+AAV-NC, SCI+AAV-NC and SCI+AAV-CLU group. Scale bar, 50 μm for original and 20 μm for enlarged pictures. Two-sided comparison; All data are mean ± SD; Error bars represent SDs; *p < 0.05, **p < 0.01, ***p < 0.001, ns, not significant, p > 0.05. See also **Supplementary Figure 6**.

Neuron-specific CLU overexpression led to significant and sustained motor function improvement. From postoperative days 7 to 28, mice receiving AAV-CLU exhibited progressively higher BMS scores compared to control virus-injected mice **(Figure 7A)**. Footprint analysis at 4 weeks corroborated this recovery, showing that CLU-overexpressing mice displayed significantly reduced hindlimb dragging and increased stride length, consistent with a plantar-stepping ability **(Figures 7B-7D)**. This functional benefit was accompanied by improved general health, as CLU overexpression substantially attenuated the significant body weight loss typically observed after SCI **(Figure S6A)**.

The functional recovery was supported by electrophysiological and histological evidence. At 4 weeks post-SCI, CLU-overexpressing mice showed a significant increase in motor evoked potential (MEP) amplitude, indicating successful conduction of nerve impulses across the lesion site, although latency remained unchanged **(Figures 7E-7G)**. Histologically, spinal cords from the AAV-CLU group exhibited superior structural preservation, with a significantly greater number of surviving NeuN-positive neurons and more Nissl-positive cells, alongside a notable reduction in tissue cavitation compared to controls** (Figures 7H-7K and S6B-S6C)**.

In summary, CLU overexpression confers comprehensive neuroprotection, enhancing neuronal survival, preserving spinal cord integrity, and ultimately promoting robust motor function recovery after SCI.

## Discussion

**Figure 8:**
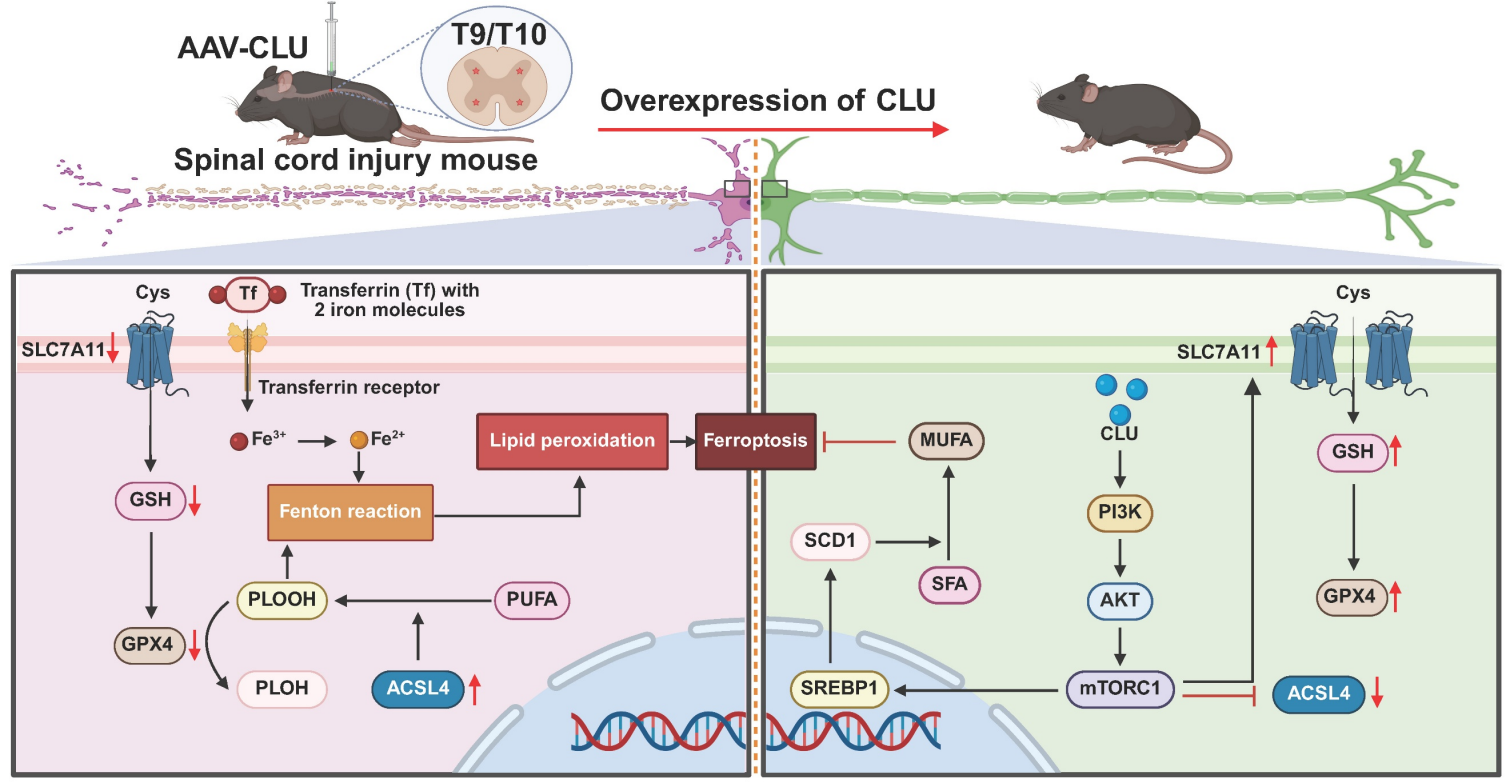
** Schematic illustration of overexpression of CLU alleviates ferroptosis after spinal cord injury by regulating lipid metabolism and antioxidant pathways.** Spinal cord injury (SCI) mice were injected with AAV-CLU at the T9/T10 spinal cord segment to induce CLU overexpression. In the absence of CLU (left panel), SCI promotes ferroptosis through decreased SLC7A11-mediated cystine uptake, reduced GSH levels, impaired GPX4 activity, increased ACSL4-driven lipid peroxidation, and iron-dependent Fenton reaction. In contrast, CLU overexpression (right panel) activates the PI3K/AKT/mTORC1 pathway, upregulates SLC7A11 and GPX4, reduces ACSL4 activity, and enhances SCD1-mediated MUFA synthesis, collectively inhibiting lipid peroxidation and ferroptosis, thereby protecting neurons after SCI.

The role of Clusterin (CLU) in programmed cell death (PCD) remains debated, with conflicting reports on its neuroprotective effects in different neurological disease models. However, its specific function in spinal cord injury (SCI), particularly concerning the critically established role of neuronal ferroptosis as a driver of secondary damage, was unclear. Our study resolves this uncertainty by demonstrating that CLU serves as a critical endogenous inhibitor of neuronal ferroptosis after SCI. We delineate a previously unrecognized mechanism: CLU activates the PI3K-AKT-mTOR signaling axis to enhance the SREBP1-SCD1-mediated synthesis of monounsaturated fatty acids (MUFAs), thereby countering lethal phospholipid peroxidation. This work not only identifies a unified pathway through which CLU confers neuroprotection but also nominates the PI3K-AKT-mTOR-SREBP1 axis as a compelling therapeutic target for ferroptosis-related CNS injuries **(Figure 8)**.

While CLU has been extensively implicated in regulating classical forms of PCD, such as apoptosis^38^-^40^ and autophagy^41^-^43^, its potential role in ferroptosis remains unexplored. Our data now position CLU as a feedback inhibitor of this process. Following SCI, a rapid, marked elevation of the ferroptosis driver ACSL4 occurred within hours, indicating the immediate initiation of lipid peroxidation. In contrast, CLU upregulation was delayed, peaking at day 14, precisely when ACSL4 levels began to decline. This dynamic profile is consistent with its characterization as a stress-responsive protein, suggesting an adaptive physiological role in the acute response to traumatic stress, followed by a return to baseline levels, potentially mediated by negative feedback or other regulatory mechanisms^44^-^47^. This hypothesis is supported by the significant co-localization of CLU with ACSL4 in neurons, implying direct regulatory crosstalk. Functionally, exogenous CLU protein confirmed this protective role, enhancing neuronal survival against ferroptosis by suppressing lipid peroxidation and shifting the expression balance of key regulators, promoting ferroptosis suppressors (GPX4, xCT) while repressing the driver ACSL4.

Previous research indicates that CLU's subcellular localization dictates its functional dichotomy^48^: nuclear CLU is often associated with pro-apoptotic effects^49^, while cytoplasmic CLU typically exhibits anti-apoptotic activity^50^. This compartmentalization underscores the critical importance of defining CLU localization within specific pathological contexts. In our study, immunofluorescence analysis of spinal cord tissue following mouse SCI revealed a significant upregulation of CLU expression specifically localized to the neuronal cytoplasm. Consistently, in an *in vitro* neuronal ferroptosis model, CLU was also upregulated and predominantly localized to the cytoplasm. Collectively, this consistent cytoplasmic localization pattern strongly supports the inference that CLU functions as a neuroprotective factor inhibiting neuronal ferroptosis in the context of SCI.

Our findings establish the activation of the PI3K-AKT-mTOR axis as the central mechanism for CLU's anti-ferroptotic effect. This discovery conceptually expands the role of this classic survival pathway, positioning it as a master regulator that integrates defense against three distinct forms of cell death: apoptosis, autophagy, and now ferroptosis. While previous studies have indeed established CLU's cytoprotection via PI3K/AKT is well-documented, such as mitigating ROS-induced apoptosis in human retinal cells 51 and promoting survival in prostate cells 52. Similarly, CLU has been shown to maintain cell viability under metabolic stress by activating PI3K/AKT/mTOR to suppress excessive autophagy^42^, our work demonstrates that CLU co-opts this very same cascade to combat the biochemically unique process of ferroptosis. This unified mechanism reveals CLU as a master coordinator of cellular resilience and elevates the PI3K-AKT-mTOR pathway as a high-value therapeutic target for multifactorial neuroprotection, capable of simultaneously mitigating multiple synergistic drivers of secondary injury in SCI.

The neuroprotective function of CLU remains controversial. For example, Han *et al.* suggested a detrimental effect, as CLU aggregation in dying neurons was associated with enhanced caspase-3 activation, and CLU knockout paradoxically reduced H-I injury by 50%^53^. In contrast, Wehrli *et al.* reported a protective role, showing that CLU overexpression mitigated ischemic damage by reducing penumbra size and promoting tissue repair after MCAO^29^. Therefore, to resolve this discrepancy and definitively assess whether CLU protects against SCI by inhibiting ferroptosis, we employed AAV-mediated, neuron-specific CLU overexpression. Our results provide conclusive evidence for its protective role: CLU overexpression comprehensively suppressed hallmark ferroptotic events, including mitochondrial damage, iron accumulation, and lipid peroxidation, while concomitantly promoting functional recovery. Furthermore, compared to transient small-molecule inhibitors (e.g., Ferrostatin-1), AAV-CLU offers the advantage of sustained, endogenous neuroprotection, underscoring its translational potential. This study proposes a promising clinical therapeutic avenue, though further experimental validation of efficacy and safety remains imperative.

Interestingly, recent work identified apolipoprotein H (APOH) as a parallel suppressor of neuronal ferroptosis by suppressing lipid peroxidation and maintaining membrane stability^54^. Given these parallels, it is plausible that CLU and APOH may participate in overlapping or cooperative pathways that modulate ferroptotic sensitivity in neurons. Exploring potential crosstalk between CLU and APOH could reveal cooperative networks regulating ferroptotic sensitivity, opening new avenues for therapeutic targeting.

Limitations of this study must be noted. The HT22 cell line does not fully replicate the complexity of mature *in vivo* neurons; the exclusive use of female mice necessitates future studies in both sexes to evaluate potential sex-dependent effects; the precise molecular interface for CLU's direct regulation of the PI3K-AKT-mTOR pathway remains to be resolved; and the current AAV delivery paradigm requires invasive injection, underscoring the need for non-invasive targeted systems (e.g., nanocarriers). Collectively, these limitations delineate clear directions for subsequent research: validating this mechanism in primary neurons and *in vivo* models of both sexes, fully mapping the CLU signaling network, and optimizing translational delivery strategies.

In summary, our study establishes CLU as a pivotal endogenous inhibitor of neuronal ferroptosis after SCI. We mechanistically link CLU's cytoprotective effect to the activation of the PI3K-AKT-mTOR-SREBP1-SCD1 signaling axis, which ultimately constrains ACSL4-mediated lipid peroxidation. This work not only resolves a key controversy regarding CLU's role in neural repair but also provides a solid experimental foundation for developing novel, pathway-targeted therapies against ferroptosis-driven central nervous system damage.

## Supplementary Material

Supplementary figures and tables.

## Abbreviations

SCI: spinal cord injury
CLU: Clusterin
PI3K: phosphoinositide 3-kinase
AKT: protein kinase B
mTOR: mammalian target of rapamycin
SREBP1: sterol regulatory element-binding protein 1
SCD1: stearoyl-CoA desaturase 1
ACSL4: acyl-CoA synthetase long-chain family member 4
GPX4: glutathione peroxidase 4
xCT: cystine/glutamate antiporter solute carrier family 7 member 11
BSCB: blood-spinal cord barrier
PUFAs: polyunsaturated fatty acids
MDA: malondialdehyde
4-HNE: 4-hydroxynonenal
PCD: programmed cell death
DFO: deferoxamine
TBI: traumatic brain injury
ROS: reactive oxygen species
MUFA: monounsaturated fatty acid
DMEM: Dulbecco's Modified Eagle Medium
FBS: fetal bovine serum
RSL3: RAS-selective-lethal-3
PBS: phosphate-buffered saline
CCK-8: Cell Counting Kit-8
cDNA: complementary DNA
RIPA: radioimmunoprecipitation assay
PFA: paraformaldehyde
H&E: hematoxylin and eosin
TEM: transmission electron microscopy
BMS: Basso Mouse Scale
MEP: motor evoked potential
DHE: dihydroethidium
DEGs: differentially expressed genes
KEGG: Kyoto Encyclopedia of Genes and Genomes
GO: Gene Ontology
SD: standard deviation
WB: Western blot
rCLU: recombinant CLU protein
Fer-1: Ferrostatin-1
RAPA: rapamycin
APOH: apolipoprotein H
AAV: adeno-associated virus
