# Supplementary material for: Clusterin Inhibits Neuronal Ferroptosis via the PI3K-AKT-mTOR-SREBP1 Axis to Promote Functional Recovery after Spinal Cord Injury
Source: Int J Biol Sci. 2026 Jan 15;22(4):1733–51. doi: 10.7150/ijbs.124291 (PMC12905574; doi:10.7150/ijbs.124291)
Supplement: Supplementary file 1 — Supplementary figures and tables. [file ijbsv22p1733s1.pdf]

Supplemental materials

Clusterin Inhibits Neuronal Ferroptosis via the PI3K-AKT-mTOR-SREBP1 Axis to Promote Functional Recovery

Yao et al.

Figures and Legends

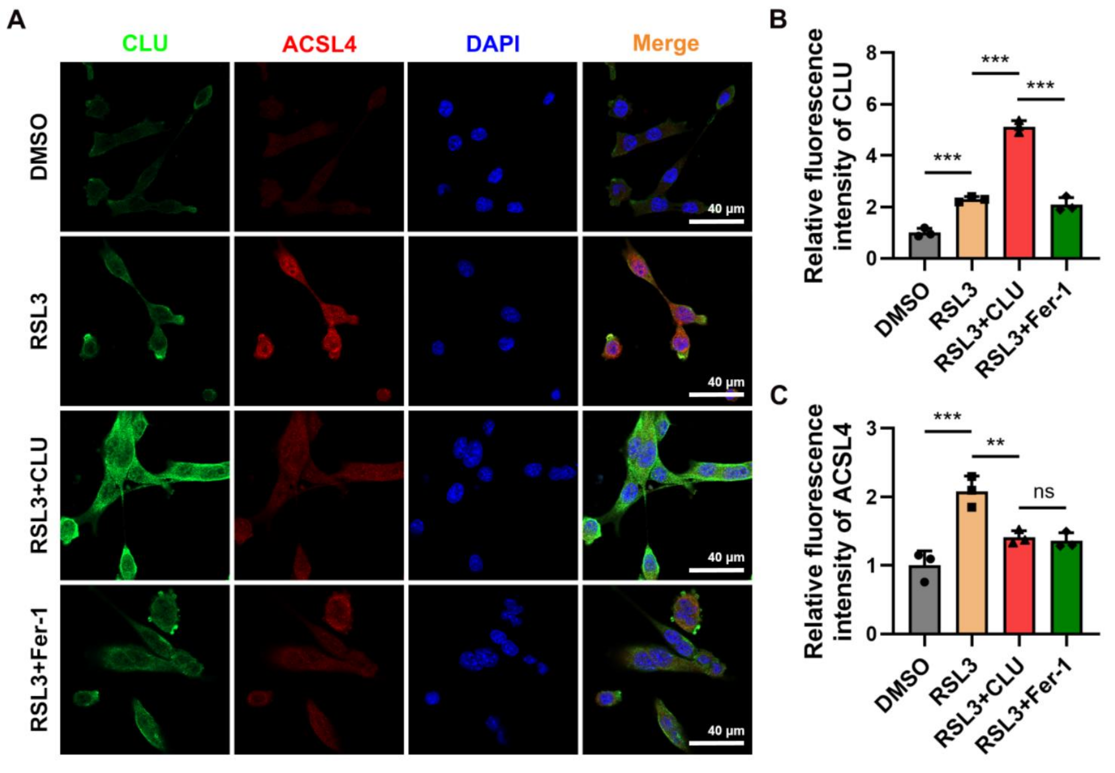

Supplementary Figure 1 | Exogenous addition of CLU protein reduced the expression of ACSL4, a marker of neuronal ferroptosis.

(A) Representative immunofluorescent staining images showing co-localization of CLU and ACSL4 in HT22 cells treated with: DMSO, RSL3 alone, RSL3 plus recombinant CLU protein, or RSL3 plus ferroptosis inhibitor Fer-1 (ferroptosis inhibitor). CLU is labeled in green, and ACSL4 in red. HT22 cells were stimulated with 5 μM RSL3 to induce ferroptosis. Scale bar, 40 μm.

(B) Quantification of fluorescence intensity of CLU in **(A)**. (n=3 biological repeats for each group; One-way ANOVA with Tukey's multiple comparisons test).

(C) Quantification of fluorescence intensity of ACSL4 in **(A)**. (n=3 biological repeats for each group; One-way ANOVA with Tukey's multiple comparisons test).

Two-sided comparison; All data are mean ± SD; Error bars represent SDs; **$p < 0.01$, ***$p < 0.001$, ns, not significant, $p > 0.05$.

See also **Figure 2**.

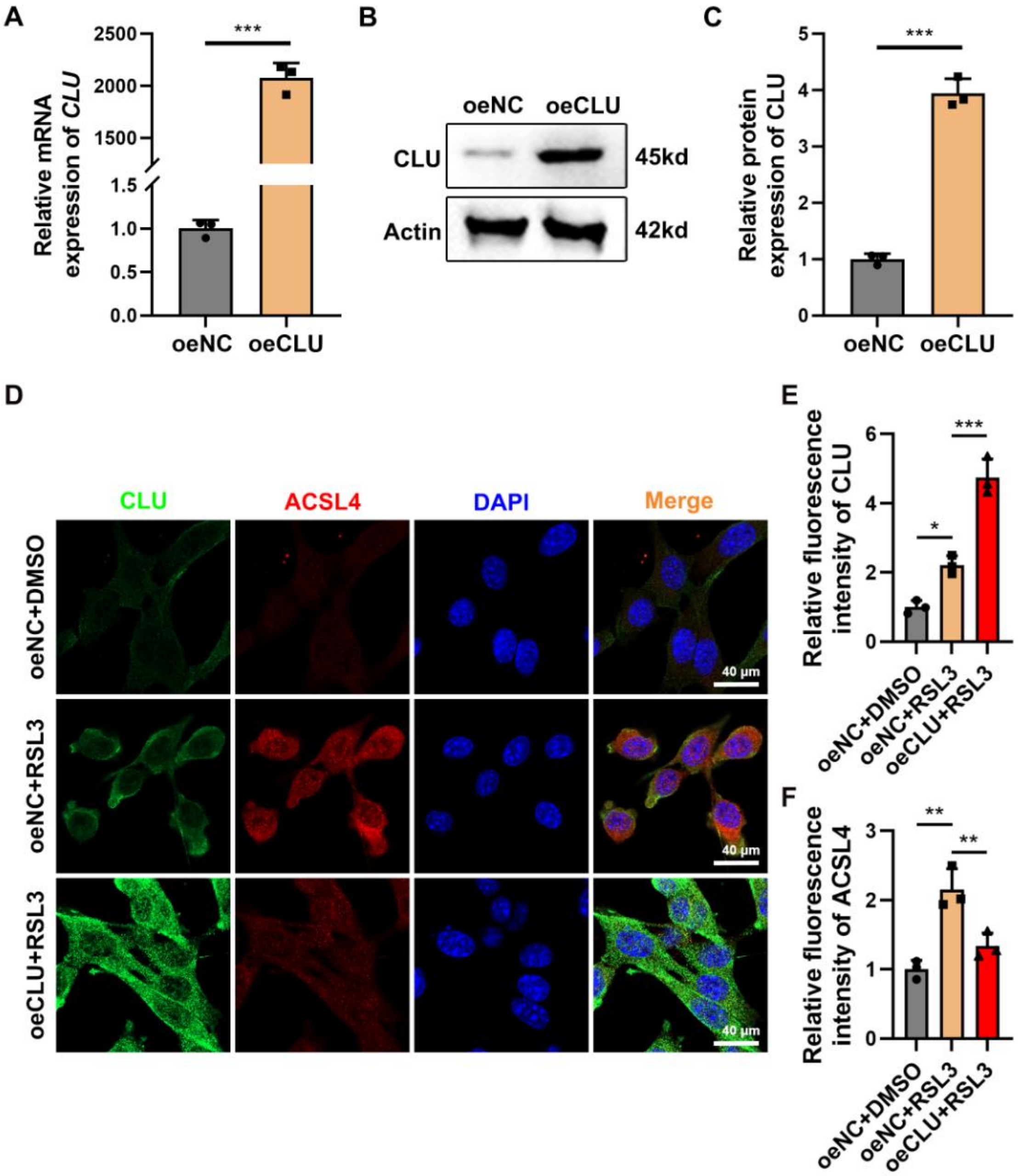

Supplementary Figure 2 | Efficiency validation of endogenous CLU overexpression and its downregulation of the neuronal ferroptosis marker ACSL4.

(A) qPCR analysis of relative mRNA expression levels of the CLU gene in the oeNC and oeCLU group. (n = 3 biological repeats for each group; Unpaired t test).

(B-C) Western blot analysis and quantification of relative CLU protein

expression level in the oeNC and oeCLU group. (n = 3 biological repeats for each group; Unpaired t test).

(D) Representative immunofluorescent staining images showing co-localization of CLU and ACSL4 in HT22 cells with different treatment: oeNC + DMSO, oeNC + RSL3, and oeCLU + RSL3. Scale bar, 40 μm.

(E) Quantification of fluorescence intensity of CLU in **(D)**. (n=3 biological repeats for each group; One-way ANOVA with Tukey's multiple comparisons test).

(F) Quantification of fluorescence intensity of ACSL4 in **(D)**. (n=3 biological repeats for each group; One-way ANOVA with Tukey's multiple comparisons test).

Two-sided comparison; All data are mean ± SD; Error bars represent SDs; *p <0.05, **p < 0.01, ***p < 0.001.

See also **Figure 3**.

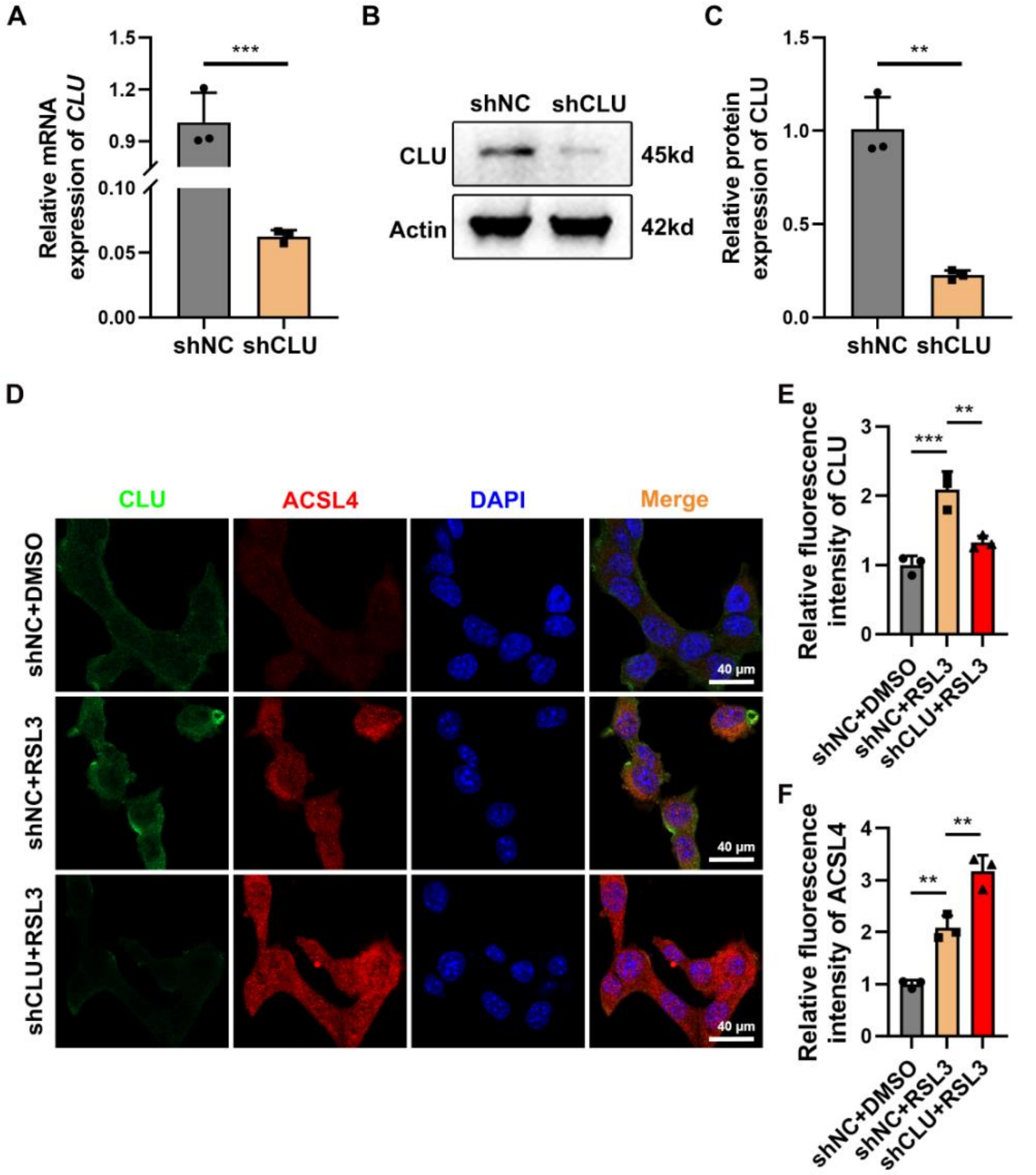

Supplementary Figure 3 | Efficiency validation of CLU knockdown and its upregulation of the neuronal ferroptosis marker ACSL4.

(A) qPCR analysis of relative mRNA expression levels of the CLU gene in the shNC and shCLU group. (n = 3 biological repeats for each group; Unpaired t test).

(B-C) Western blot analysis and quantification of relative CLU protein

expression level in the shNC and shCLU group. (n = 3 biological repeats for each group; Unpaired t test).

(D) Representative immunofluorescent staining images showing co-localization of CLU and ACSL4 in HT22 cells with different treatment: shNC + DMSO, shNC + RSL3, and shCLU + RSL3. Scale bar, 40 μm.

(E) Quantification of fluorescence intensity of CLU in **(D)**. (n=3 biological repeats for each group; One-way ANOVA with Tukey's multiple comparisons test).

(F) Quantification of fluorescence intensity of ACSL4 in **(D)**. (n=3 biological repeats for each group; One-way ANOVA with Tukey's multiple comparisons test).

Two-sided comparison; All data are mean ± SD; Error bars represent SDs; **$p < 0.01$, ***$p < 0.001$.

See also **Figure 4**.

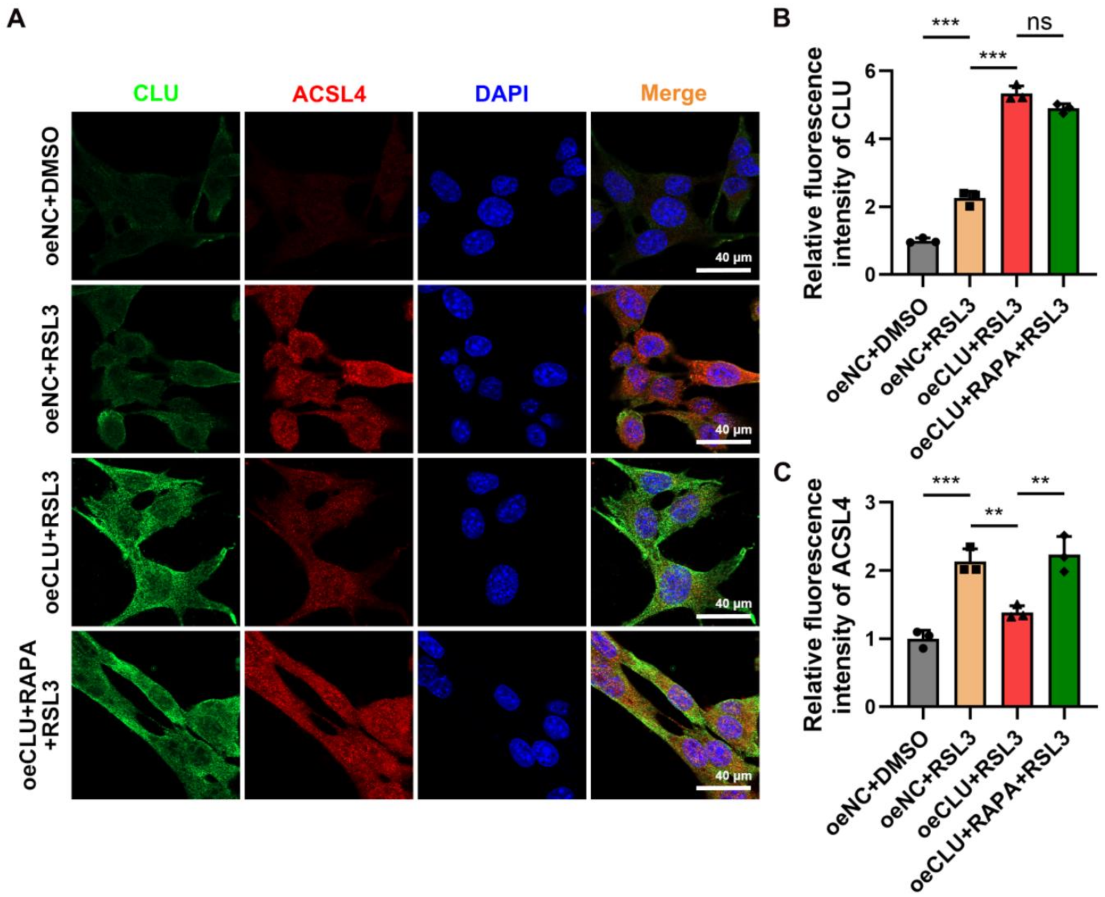

Supplementary Figure 4 | CLU reduced the expression of ACSL4 by activating the PI3K-AKT-mTOR pathway in vitro.

(A) Representative immunofluorescent staining images showing co-localization of CLU and ACSL4 in HT22 with different treatment groups: oeNC (empty vector), oeNC+RSL3 (ferroptosis inducer), oeCLU (CLU overexpression) +RSL3, oeCLU+RSL3+RAPA (mTOR inhibitor). Scale bar, 40 μm.

(B) Quantification of fluorescence intensity of CLU in **(A)**. (n=3 biological repeats for each group; One-way ANOVA with Tukey's multiple comparisons test).

(C) Quantification of fluorescence intensity of ACSL4 in **(A)**. (n=3 biological repeats for each group; One-way ANOVA with Tukey's multiple comparisons test).

Two-sided comparison; All data are mean ± SD; Error bars represent SDs; **$p < 0.01$, ***$p < 0.001$, ns, not significant, $p > 0.05$.

See also **Figure 5**.

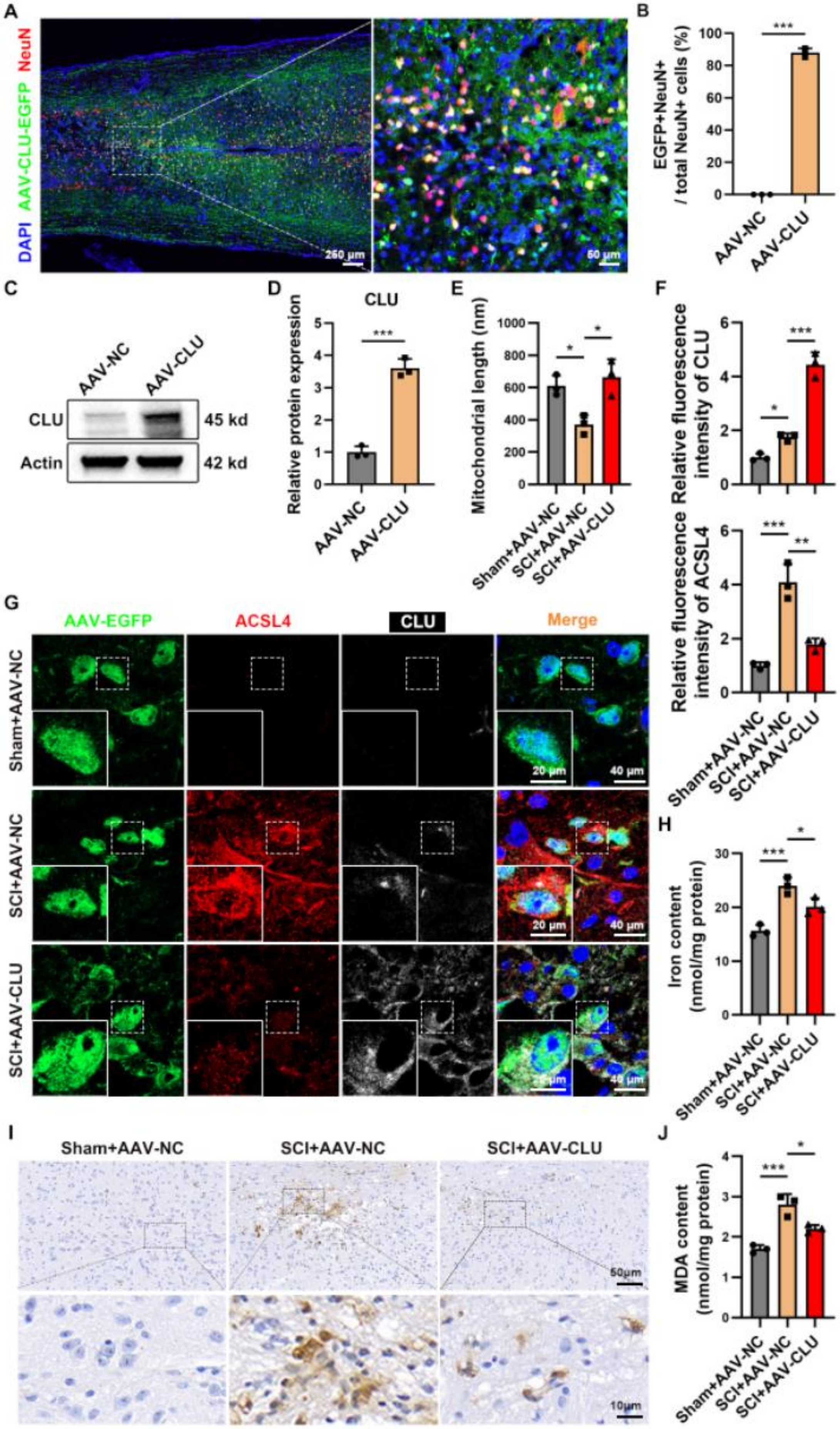
A
DAPI AAV-CLU-EGFP NeuN
250 μm
50 μm
B
EGFP+NeuN+ / total NeuN+ cells (%)
AAV-NC
AAV-CLU
C
AAV-NC
AAV-CLU
CLU
Actin
45 kd
42 kd
D
CLU
Relative protein expression
E
Mitochondrial length (nm)
Sham+AAV-NC
SCI+AAV-NC
SCI+AAV-CLU
F
Relative fluorescence intensity of CLU
Relative fluorescence intensity of ACSL4
G
AAV-EGFP
ACSL4
CLU
Merge
20 μm
40 μm
H
Iron content (nmol/mg protein)
I
50μm
10μm
J
MDA content (nmol/mg protein)

1

Supplementary Figure 5 | In vivo validation of CLU overexpression efficiency and ferroptosis-related phenotypes.

(A) Representative immunofluorescent staining images of healthy control spinal cord tissue showing co-staining of AAV-CLU-EGFP (green, marking CLU overexpression) and NeuN (red, neuronal marker), demonstrating neuronal CLU overexpression in vivo. Scale bar, 250 μm for original and 50 μm for enlarged pictures.

(B) Quantification of the proportion of $EGFP^{+}NeuN^{+}$ cells in total $NeuN^{+}$ cells of spinal cord tissue in **(A)**. (n = 3 biological repeats for each group; Unpaired t test).

(C-D) Western blot analysis and quantification of relative CLU protein expression level in the spinal cord tissues of mice from AAV-NC and AAV-CLU group. (n = 3 biological repeats for each group; Unpaired t test).

(E) Quantification of the average mitochondrial length (nanometer, nm) in **Figure 6E**. (n=3 biological repeats for each group; One-way ANOVA with Tukey's multiple comparisons test).

(F) Quantification of fluorescence intensity of CLU and ACSL4 in **(G)**. (n=3 biological repeats for each group; One-way ANOVA with Tukey's multiple comparisons test).

(G) Representative immunofluorescent staining images of AAV-EGFP (green, marking neurons), CLU (white) and ACSL4 (red, ferroptosis marker) in the spinal cord lesions at 7 dpi of mice from Sham+AAV-NC, SCI+AAV-NC and

SCI+AAV-CLU group. Scale bar, 40 μm for original and 20 μm for enlarged pictures.

(H) Quantification of intracellular total iron ion levels in the spinal cord tissues of mice from Sham+AAV-NC, SCI+AAV-NC and SCI+AAV-CLU group. (n=3 biological repeats for each group; One-way ANOVA with Tukey's multiple comparisons test).

(I) Representative images of Prussian blue staining combined with DAB enhancement in the spinal cord lesions at 7 dpi of mice from Sham+AAV-NC, SCI+AAV-NC and SCI+AAV-CLU group. Scale bar, 50 μm for original and 10 μm for enlarged pictures.

(J) Quantification of intracellular Malondialdehyde (MDA) levels in the spinal cord tissues of mice from Sham+AAV-NC, SCI+AAV-NC and SCI+AAV-CLU group. (n=3 biological repeats for each group; One-way ANOVA with Tukey's multiple comparisons test).

Two-sided comparison; All data are mean ± SD; Error bars represent SDs; $*p < 0.05$, $**p < 0.01$, $***p < 0.001$.

See also **Figure 6**.

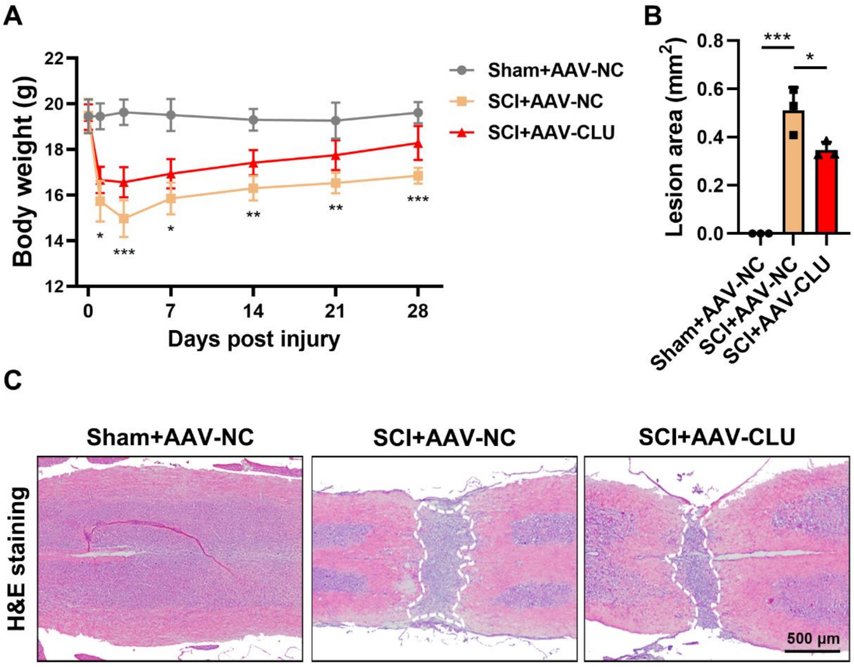

Supplementary Figure 6 | Overexpression of CLU in vivo reduces lesion area after spinal cord injury.

(A) Measurement of body weight on day 0, 1, 3, 7, 14, 21, and 28 post injury (dpi) in mice from Sham+AAV-NC, SCI+AAV-NC and SCI+AAV-CLU group. (n = 6 biological repeats for each group; Two-way ANOVA with Tukey's multiple comparisons test).

(B-C) Representative images of H&E staining and quantification of lesion area at 28 dpi of mice from Sham+AAV-NC, SCI+AAV-NC, and SCI+AAV-CLU group. Scale bar, 500 μm. (n=3 biological repeats for each group; One-way ANOVA with Tukey's multiple comparisons test).

Two-sided comparison; All data are mean ± SD; Error bars represent SDs; *p <0.05, **p < 0.01, ***p < 0.001.

See also **Figure 7**.

Supplementary Table 1 | Primer used during PCR. Related to Experimental Procedures.

Gene	Sequences (5′ to 3′)
CLU	Forward: 5’-AGCAGGAGGTCTCTGACAATG-3’
	Reverse: 5’- GGCTTCCTCTAAACTGTTGAGC-3’
Actin	Forward: 5’- AAATCGTGCGTGACATCAAAGA-3’
	Reverse:5’- GCCATCTCCTGCTCGAAGTC-3’
GPX4	Forward: 5’- CCGTCTGAGCCGCTTACTTA-3’
	Reverse: 5’- GTGACGATGCACACGAAACC-3’
xCT	Forward: 5’- AATACGGAGCCTTCCACGAG-3’
	Reverse: 5’- CTCCAGGGGCAGTCAGTTAG-3’
ACSL4	Forward: 5’- GCACCTTCGACTCAGATCACA-3’
	Reverse: 5’- GAAGCCAGCAATAAAGTACACAGA-3’

Supplementary Table 2 | Primary and secondary antibodies.

Product	Catalogue Number	Supplier
Primary antibody:		
WB:		
Anti-ACSL4	38493	Cell Signaling Technology
Anti-Clusterin	sc-166907	Santa Cruz
Anti-GPX4	ab125066	Abcam
Anti-xCT	ab307601	Abcam
Anti-4HNE	ab46545	Abcam
Anti-PI3K	4249	Cell Signaling Technology
Anti-p-PI3K	4228	Cell Signaling Technology
Anti-AKT	4691	Cell Signaling Technology
Anti-p-AKT	4060	Cell Signaling Technology
Anti-mTOR	2972	Cell Signaling Technology
Anti-p-mTOR	2971	Cell Signaling Technology
Anti-SREBP-1	sc-13551	Santa Cruz
Anti-SCD1	sc-515844	Santa Cruz
Anti-Actin	AC026	Abclonal
IF:		
Anti-ACSL4	ab155282	Abcam
Anti-CLU	ab79280	Abcam
Anti-CLU	AF2747	R&D
Anti-GFAP	ab53554	Abcam
Anti-NeuN	ab104224	Abcam
Anti-NeuN	ab177487	Abcam
Secondary antibody:		
WB:		
Anti-mouse IgG, HRP-linked Antibody	7076	Cell Signaling Technology
Anti-rabbit IgG, HRP-linked Antibody	7074	Cell Signaling Technology
IF:		
Donkey Anti-Mouse IgG H&L (Alexa Fluor® 488)	ab150105	Abcam
Donkey Anti-Mouse IgG H&L (Alexa Fluor® 555)	ab150106	Abcam
Donkey Anti-Rabbit IgG H&L (Alexa Fluor® 555)	ab150074	Abcam
Donkey Anti-Mouse IgG H&L (Alexa Fluor® 647)	ab150107	Abcam
Goat Anti-Rabbit IgG H&L (Alexa Fluor® 647)	ab150079	Abcam
Donkey Anti-Goat IgG H&L (Alexa Fluor® 647)	ab150131	Abcam

Supplementary Table 3 | Key resource table.

Product	Catalogue Number	Supplier
Chemicals, peptides, and recombinant proteins		
DMSO	D2605	Sigma-Aldrich
Triton X-100	1139ML100	Biofroxx
Trizol reagent	T9424	Sigma-aldrich
RIPA Lysis Buffer	P0013B	Beyotime
Mounting Medium with DAPI	ab104139	Abcam
Hoechst 33342	C1029	Beyotime
Isoflurane	R510-22	RWD
Tribromethanol	Sigma-Aldrich	Cat#: T48402
FBS	10099-141	Invitrogen
PBS	10010023	Gibco
DMEM	C11995500BT	Gibco
TRYPSIN 0.25% EDTA	25200072	Invitrogen
Penicillin-Streptomycin Solution	EH80010	eLGbio
Bovine serum albumin	A34787	Invitrogen
Fer-1	HY-100579	MCE
Rapamycin	HY-10219	MCE
BODIPY 581/591 C11	C1022	Beyotime
FerroOrange	HY-D1913	MCE
RSL3	HY-100218A	MCE
Recombinant Mouse Clusterin Protein	50485-M08H	Sino Biological
Critical commercial assays		
StarScript II First-strand cDNA Synthesis Kit-II	A214	GenStar
2× RealStar Fast SYBR qPCR Mix	A301	GenStar
BCA Protein Assay Kit	23227	Thermo Fisher Scientific
SDS-PAGE Gel Preparation kit	P0012A	Beyotime
Cell Counting Kit-8	GK10001	GLPBIO
MDA Assay Kit	S0131S	Beyotime
Iron Content Colorimetric Assay Kit	E1042	Applygen
DHE (Dihydroethidium) Assay Kit	ab236206	Abcam
H&E Staining Kit	G1005	Servicebio
Nissl Staining Kit	G1036	Servicebio
Prussian blue Staining Kit	G1029	Servicebio
Experimental models: Organisms/strains		
Ht22	CL-0697	Procell
Software and algorithms		
ZEN (v3.1)	http://www.zeiss.com.cn/	Zeiss

GraphPad Prism (v10)	https://www.graphpad-prism.cn/	Graphpad
Adobe Photoshop (v2024)	N/A	Adobe
ImageJ (v1.54g)	https://imagej.net/ij/	NIH
R (v 4.4.0)	https://www.r-project.org	The R Foundation
Seurat (v5.1.0)	https://satijalab.org/seurat/index.html	N/A

Supplementary Table 4 | Target sequence of shRNAs.

Name	Sequences (5′ to 3′)
shNC	CCTAAGGTTAAGTCGCCCTCG
shCLU	GCAGGAGGTCTCTGACAAT
